# Platelets mirror changes in the frontal lobe antioxidant system in Alzheimer's disease

**DOI:** 10.1002/alz.70117

**Published:** 2025-04-06

**Authors:** Huriye Ercan, Christina Maria Reumiller, Jacqueline Mühlberger, Felicia Hsu, Georg Johannes Schmidt, Ellen Umlauf, Ingrid Miller, Eduard Rappold, Johannes Attems, Rudolf Oehler, Maria Zellner

**Affiliations:** ^1^ Institute of Vascular Biology and Thrombosis Research Centre for Physiology and Pharmacology Medical University of Vienna Vienna Austria; ^2^ Department of Clinical Pharmacology Medical University of Vienna Vienna Austria; ^3^ Department of Biological Sciences and Pathobiology University of Veterinary Medicine Vienna Vienna Austria; ^4^ Translational and Clinical Research Institute Campus for Ageing and Vitality Newcastle University Newcastle UK; ^5^ Department of General Surgery Division of Visceral Surgery Medical University of Vienna Vienna Austria

**Keywords:** Alzheimer's disease, antioxidant system, blood biomarker, frontal lobe, mild cognitive impairment, platelets

## Abstract

**INTRODUCTION:**

Blood biomarkers reflecting Alzheimer's disease (AD) pathophysiology can improve diagnosis and treatment.

**METHODS:**

We applied top‐down proteomics to compare frontal lobe from 17 AD cases and 11 controls to blood platelets from a second independent study group of 124 AD patients, 61 with mild cognitive impairment (MCI), and 168 controls. Findings were immunologically validated.

**RESULTS:**

Sixty AD‐associated proteoforms were identified in frontal lobe, with 26 identically represented in platelets. Validation in platelet samples confirmed elevated glutathione S‐transferase omega 1 (GSTO1) levels linked to single nucleotide polymorphism (SNP) rs4925 and increased superoxide dismutase 1 (SOD1) levels in AD. Bioinformatics revealed copper chaperone for superoxide dismutase (CCS) and glutathione peroxidase 1 (GPX1) as integral partners of these antioxidant enzymes. Both were detected to be reduced in frontal lobes and platelets in AD. SOD1 and CCS are already changed in MCI.

**DISCUSSION:**

These four novel blood biomarkers, integrated with traditional AD biomarkers, may facilitate patient risk assessment and treatment, with SOD1 and CCS alterations in MCI offering early diagnostic potential.

**Highlights:**

- Platelets mirror several Alzheimer's disease (AD)–dependent neuronal changes, valuable for blood tests.
- As a start, 60 AD‐associated frontal lobe proteins were identified by top‐down proteomics.
- Fifty percent of these 60 AD‐affected brain proteins are represented identically in platelets.
- Among these, glutathione S‐transferase omega 1 (GSTO1), superoxide dismutase 1 (SOD1), copper chaperone for superoxide dismutase (CCS), and glutathione peroxidase 1 (GPX1) are identically AD related in brain and platelets.
- SOD1 and its crucial activating partner CCS are altered in the platelets of patients with mild cognitive impairment.

## 1 BACKGROUND

The pathogenesis of Alzheimer's disease (AD) is characterized by several hallmarks, including abnormal processing of the amyloid precursor protein (APP), resulting in the deposition of amyloid beta (Aβ),^1^, ^2^ and the hyperphosphorylation of the tau protein (p‐tau), leading to the formation of neurofibrillary tangles in the brain.^3^ These pathological changes arise from alterations in the post‐translational modifications (PTMs) of these proteins. PTMs, or genetic variations, such as the AD‐associated single nucleotide polymorphism (single nucleotide polymorphism [SNP]) apolipoprotein E (*APOE*) ε4, can modify protein functionality or lead to its dysfunction. Collectively, these alterations are referred to as proteoforms of specific proteins.^4^

With respect to sporadic AD, the initial molecular causes of Aβ and tau pathology as well as of the accelerated neurodegeneration are still largely unknown.^5^ Emerging evidence suggests that oxidative stress linked to aging exacerbates neurodegeneration and cognitive decline in AD.^6^ Early detection of AD requires a versatile approach, including biomarkers to uncover these stressors and the accumulation of Aβ and p‐tau proteoforms.

Understanding of the molecular changes influencing AD onset and progression is crucial. Inexpensive blood tests are needed to routinely detect AD's biochemical signature. Detecting Aβ and p‐tau proteoforms in plasma allows reliable peripheral detection of AD‐related changes.^7^, ^8^ Elevated plasma glial fibrillary acidic protein (GFAP), a marker of astroglial activation in the brain, has also been identified as a helpful blood biomarker for AD.^9^ Despite these developments, further blood‐based biomarkers are needed to causally reflect the complexity of AD pathogenesis.

Platelets share several biochemical properties with neurons and are promising tools for discovering peripheral cellular disorders linked to AD.^10^, ^11^ First, platelets contain large amounts of APP and express α‐, β‐, and γ‐secretases, which in turn generate several AD‐related changes in APP cleavage proteoforms in the circulation.^12^, ^13^, ^14^ Second, they store neurotransmitter‐degrading enzymes such as monoamine oxidase B (MAOB), the concentration of which increases in both platelets and the brains of patients with AD,^10^, ^15^, ^16^ leading to increased oxidative deamination of monoamines such as dopamine. Third, platelets, like neurons, are subject to enhanced oxidative stress with increasing age, a further mechanism that accelerates AD.^17^ Systemic alterations in AD mirrored in a homogenous platelet population may directly reflect neuronal cell–associated AD‐related changes, leaving out degenerative heterogeneous tissue changes occurring in affected brain regions. Compared to brain tissue, platelets are easily accessible and represent an attractive protein biomarker source for studying AD‐related pathological changes.^11^

Sporadic AD is assumed to result from an interplay of age, lifestyle, environment, and genetic factors, all reflected in complex changes of proteins and their proteoforms.^18^ Many proteomic studies have investigated these changes in various brain regions, cerebrospinal fluid (CSF), and plasma. Some studies have also examined the platelets of patients with AD, identifying AD‐related protein changes.^15^, ^19^, ^20^, ^21^, ^22^ However, no unbiased proteomic study has directly and comprehensively compared brain and platelet samples to identify identical proteoform alterations in AD.

Bottom‐up (shotgun) proteomics, used predominantly today, has severe limitations, as it requires digesting proteins into peptides, resulting in a loss of proteoform‐specific information within an unassignable mix of peptide fragments. In contrast, top‐down proteomics analyzes the intact proteome without proteolytic digestion, thereby preserving proteoform data, the genuine mechanistic players, and thus valuable biomarkers.^23^ The drawback of bottom‐up proteomics in detecting peptides alone likely contributes to false‐negative or false‐positive results, which only indicate alterations in canonical proteins, not their critical proteoforms. The top‐down proteomics approach, two‐dimensional difference gel electrophoresis (2D‐DIGE), remains the most effective technology for comprehensively analyzing intact proteomes and identifying critical proteoforms in complex samples and across different proteomes.^24^

This study aims to identify protein biomarkers in platelets that identically mirror AD‐related changes in affected brain regions. Differentially labeled protein extracts from the frontal lobe and platelets were directly compared using an integrative top‐down proteomic approach by 2D‐DIGE to detect identical AD‐related brain proteoforms in platelets. Subsequently, these brain‐identical AD‐related proteoforms were quantified in platelet samples from patients with AD and patients with mild cognitive impairment (MCI) and age‐ and sex‐matched cognitively healthy controls using the same technical 2D‐DIGE settings to investigate their association with cognitive decline.

## 2 METHODS

### 2.1 Frontal lobe tissue samples

Frozen brain tissue samples of the frontal lobe (BA9) were provided by the Newcastle Brain Tissue Resource (NBTR), Newcastle University, UK, in accordance with the approval of the joint Ethics Committee of Newcastle and North Tyneside Health Authority, and NBTR brain banking procedures were followed. The neuropathological diagnostic assessment was performed according to internationally accepted criteria, including Thal stages of Aβ deposition,^25^ Braak neurofibrillary tangle stages,^26^ Consortium to Establish a Registry for Alzheimer's Disease (CERAD) criteria,^27^ National Institute on Aging–Alzheimer's Association (NIA‐AA) criteria,^28^ Braak Lewy body stages,^29^ and Newcastle McKeith criteria.^30^ The study cohort consisted of 17 AD cases, showing clinical dementia and high AD neuropathologic changes according to the NIA‐AA criteria, lacking other significant neurodegenerative pathologies, and 11 controls displaying neither cognitive impairment nor significant neurodegenerative pathology.

On average, brain tissue samples were snap‐frozen 35 h post‐mortem (min 5 h/max 95 h) and were stored at −80°C until further use. Demographic details are shown in Table 1A.

**TABLE 1:** Demographic details of the study cohort.

A of frontal lobe tissue samples			
	AD	Control	*p*‐value
***N***	17	11	
**Gender**			0.564
Women, *n* (%)	7 (38)	6 (55)	
Men, *n* (%)	10 (63)	5 (46)	
**Age**, y, mean ±	82.1 ± 8	76.3 ± 10	0.098
**Postmortem delay,** h	29.7 ± 20	44.4 ± 24	0.132
***APOE* ε4/ε4,** *n* (%)	2 (12)	0 (0)	
***APOE* ε4/ε** ^a^, *n* (%)	13 (75)^*^	1 (9.1)	0.013
***GSTO1*** (rs4925)			0.930
^b^CC, *n* (%)	7 (43.8)	5 (45.5)	
^c^CA, *n* (%)	8 (50.0)	4 (36.4)	
^d^AA, *n* (%)	1 (6.3)	2 (18.2)	

B of platelet samples				
	AD	MCI	Control	*p*‐value
***N***	124	61	168	
**Gender**				0.249
Women, *n* (%)	82 (66)	46 (75)	107 (64)	
Men, *n* (%)	42 (34)	15 (25)	61 (36)	
**Age**, y	79.7 ± 7	77.2 ± 8	78.7 ± 8	0.163
**Education**, y	11.3 ± 3^*^	11.7 ± 2	12.7 ± 3	0.013
**MMSE**	14.8 ± 8^***^	27.6 ± 1^***^	28.9 ± 1	6.3E‐37
**Platelets**, ×10^9^/L	228 ± 84	224 ± 65	228 ± 61	0.523
***APOE* ε4/ε4,** *n* (%)	25 (20)^***^	2 (3)	1 (1)	7.8E‐15
***APOE* ε4/ε** ^a^, *n* (%)	82 (66)^***^	17 (28)^***^	26 (16)	4.1E‐22
***GSTO1*** *(rs4925)*				0.266
^b^CC, *n* (%)	68 (54.8)	35 (57.4)	74 (44.0)	
^c^CA, *n* (%)	46 (37.1)	20 (32.8)	74 (44.0)	
^d^AA, *n* (%)	10 (8.1)	6 (9.8)	20 (11.9)	

*Note*: Samples derived from (a) frontal lobe tissue and (b) blood of AD, MCI, and control subjects were genotyped. Platelet counts were determined in whole blood anti‐coagulated with EDTA. Genotyping was performed with genomic DNA extracted from brain or EDTA whole blood. Unless otherwise stated, values represent mean ± SD. The Kruskal–Wallis test was used to compare continuous variables among multiple groups (AD, MCI, and controls). The Mann–Whitney *U* test was applied for pairwise comparisons of continuous variables to the control group. Cross tables for multiple groups and the chi‐square test were used for pairwise comparison of categorical variables.

Abbreviations: AD, Alzheimer's disease; *APOE*, apolipoprotein E; EDTA, ethylenediaminetetraacetic acid GSTO1, glutathione S‐transferase omega 1; MCI, mild cognitive impairment; MMSE, Mini‐Mental State Examination; SD, standard deviation.

* *p* < 0.05, significant difference compared to controls.

*** *p* < 0.001, highly significant difference compared to controls.

No asterisk *p* ≥ 0.05, not statistically significant compared to controls.

^a^ Percentage of *APOE* ε4 positivity (homo‐ or heterozygous).

^b^ GSTO1*CC homozygous major *GSTO1* allele carrier (Ala140/Ala140).

^c^ GSTO1*CA heterozygous major *GSTO1* allele carrier (Ala140/Asp140).

^d^ GSTO1*AA homozygous minor *GSTO1* allele carrier (Asp140/Asp140).

### 2.2 Population demographics of platelet sample cohort

All study participants were recruited in Austria and are of Caucasian descent. Patients with AD were from geriatric, retirement, and nursing homes. Cognitively healthy controls and patients with MCI comprise a selection of spouses and caregivers of AD patients and of residents in retirement homes. The Ethics Committee of Vienna approved the study protocol (EK 04‐070‐0604 and EK 09‐219‐1209), and the trial was conducted in accordance with the Declaration of Helsinki. All study participants or their trustees signed an informed consent before study entry. Only non‐smoking individuals were recruited, and none of the study participants received any antipsychotic drugs or antidepressants. The participants underwent cognitive neuropsychological evaluation on the day of blood sampling using the Mini‐Mental State Examination (MMSE) and the CERAD test battery.^31^ Due to advanced AD, some patients were evaluated by the MMSE only. A clinical examination, including an anamnesis, was performed to exclude any other systemic disorders that might affect the cognitive status. The platelet proteomic data of the present study derive from 2D‐DIGE analyses of 62 AD patients and 63 age‐ and sex‐matched controls, first published in 2014.^22^ This study group was expanded to 86 AD patients, 30 MCI, and 86 controls for a follow‐up AD biomarker work in 2017.^32^ This collective was further enlarged to 124 AD patients, 61 MCI, and 168 age‐ and sex‐matched cognitively healthy controls for the present study.

**RESEARCH IN CONTEXT**

1. **Systematic review**: Our thorough literature search in PubMed revealed substantial evidence that platelets are valuable biomarker sources for AD and blood‐based diagnostic tests. Since 1980, studies have shown parallel changes in monoamine oxidase B (MAOB), amyloid precursor protein (APP), and oxidative stress in both the platelets and brains of patients with Alzheimer's disease (AD). Yet, no comprehensive studies exist on further identical protein changes in brain and platelets in AD.
2. **Interpretation**: Our inter‐tissue proteomic analysis found that in AD, four proteins crucial for antioxidant protection (superoxide dismutase 1 [SOD1], copper chaperone for superoxide dismutase [CCS], glutathione peroxidase 1 [GPX1], and glutathione S‐transferase omega 1 [GSTO1]) are similarly altered in both frontal lobe and platelets. These changes may indicate a weakened response to oxidative stress in AD‐affected brain regions, mirrored in the blood.
3. **Future directions**: Future studies should explore how these antioxidative alterations affect oxidative stress in neurons and AD‐related pathophysiology and if targeting these mechanisms can help. In addition, these new blood biomarkers may enhance the diagnostic value and causality in combination with traditional AD markers.

To exclude any other brain pathology accounting for cognitive impairment, such as stroke or tumors, the entire group of clinically suspected AD patients underwent structural brain scanning using magnetic resonance imaging (MRI). Finally, diagnosis of probable AD was made by a physician and a psychologist according to the criteria by the U.S. National Institute of Neurological and Communicative Disorders and Stroke and the Alzheimer's Disease and Related Disorders Association.^33^ Approximately 20% of the recruited AD patients received acetylcholinesterase inhibitors (e.g., donepezil) or *N*‐methyl‐d‐aspartate (NMDA) receptor antagonists (e.g., memantine). AD‐related medication was distributed equally between genders. Patients with MCI were diagnosed by a psychologist and a physician according to the guidelines of the consensus conference in Stockholm.^34^ Clinical criteria for MCI were MMSE ≥24, not demented, intact activities of daily living, and impairment in at least two domains of memory of the CERAD using diagnostic comprehensive criteria.^35^ None of the patients with MCI received AD‐related medication. Controls were classified as cognitively healthy by a physician and psychologist when the MMSE was ≥26 and the CERAD test battery corresponded to age‐appropriate norms in all cognitive domains.^31^ Demographic details are shown in Table 1B.

### 2.3 Cryo‐homogenization and protein extraction of frontal lobe samples

Snap‐frozen frontal lobe brain samples weighing 30–95 mg were individually placed into pre‐cooled 8 cm × 15 cm sterile plastic bags and finely pulverized using a pre‐cooled hammer under liquid nitrogen according to a published protocol.^36^ These cryo‐homogenized samples were stored at −80°C until protein extraction was performed with 500 µL of re‐solubilization buffer containing 7 M urea, 2 M thiourea, 4% 3‐[(3‐Cholamidopropyl)dimethylammonio]‐1‐propanesulfonate (CHAPS), and 20 mM Tris‐HCl pH 8.5. Thiourea prevents proteolytic degradation of the sample.^37^ A sonication step using an ice‐cooled ultrasonic bath was followed to facilitate extraction and dissolution. Subsequently, the samples were agitated on a shaker for 30 min at 4°C and 550 rpm, followed by centrifugation for 10 min at 4°C and 15,000 × *g*. The supernatant was transferred into a new 1.5 mL reaction tube, thereby carefully avoiding catching the loose pellet or absorbing particles of the lipid layer on the top of the aqueous phase.

### 2.4 Blood sampling, platelet isolation, and protein extraction from alive study cohorts

Blood was collected from the antecubital vein using a 21‐gauge needle without stasis into Vacutainer tubes (Greiner Bio‐One GmbH, Kremsmünster, Austria) containing 0.129 mol/L sodium citrate as an anticoagulant. The first obtained tube was discarded. Platelets were subsequently isolated as described previously.^22^ Briefly, whole blood was centrifuged for 20 min at 120 × *g* at room temperature. Resulting platelet‐rich plasma (PRP) was gel‐filtered to separate platelets from plasma proteins using Sepharose 2B (GE Healthcare, Uppsala, Sweden) packed columns (Econo‐Pac 15 mm diameter, Bio Rad, Hercules, USA). Gel‐filtered platelets (GFPs) were eluted in calcium‐ and magnesium‐free Dulbecco's phosphate‐buffered saline (PBS; Thermo Fisher Scientific, Paisley, Scotland, UK). Platelet counts in GFP fractions were assessed with a Sysmex KX‐21N hematological analyzer (Sysmex Kobe, Japan). Immediately after isolation, the platelet proteins were extracted by addition of a 6.1 N trichloroacetic acid (TCA) solution containing 80 mM dithiothreitol (DTT), (Roche Diagnostics, Mannheim, Germany) at a ratio of one volume TCA solution to three volumes GFP suspension. After incubation for 1 h at 4°C, platelet proteins were pelleted by centrifugation for 10 min at 20,000 × *g* and 4°C. Pellets were washed four times with acetone (Merck, Darmstadt, Germany) containing 20 mM DTT. Prior to re‐solubilization in urea‐sample buffer (7 M urea, 2 M thiourea, 4% CHAPS, 20 mM Tris‐HCl, pH 8.5), the platelet samples were washed once with acetone without DTT. For re‐solubilization, the platelet pellets were then incubated overnight at 4°C with stirring at 800 rpm.

### 2.5 Determination of protein concentrations of frontal lobe and platelet samples

The protein content of solubilized frontal lobe and platelet samples was determined immediately using the Coomassie Plus assay kit (Thermo Fisher Scientific, Rockford, Illinois, USA). An internal standard sample for the 2D‐DIGE analyses was generated by combining equal amounts of protein from the respective samples used in the frontal lobe or platelet 2D‐DIGE study. Each of the platelet and frontal lobe protein samples, along with the IS, was divided into aliquots containing 50 µg of protein and stored at −80°C.

### 2.6 Top‐down proteome analysis of frontal lobe and platelet sample by 2D‐DIGE

Prior to isoelectric focusing (IEF), 12 µg of protein samples from the frontal lobe or platelets was labeled with fluorescent cyanine dyes (5 pmol of CyDyes per µg of protein; GE Healthcare, Uppsala, Sweden) in the re‐solubilization buffer containing 7 M urea, 2 M thiourea, 4% CHAPS, and 20 mM Tris‐HCl (pH 8.5), following a protocol described previously.^19^ The labeling reaction was terminated by adding 5 mM lysine.

Samples from the two respective study groups, labeled alternately with Cy3 or Cy5, were combined with the IS labeled with Cy2 (also 12 µg). The final volume of this pool was adjusted to 250 µL using a rehydration buffer containing 7 M urea, 2 M thiourea, and 4% CHAPS, and supplemented to a final concentration of 70 mM DTT and 0.5% pH 4–7 ampholytes (Serva, Heidelberg, Germany). IPG‐Dry‐Strips (24 cm, pH 4–7; GE Healthcare) were then passively rehydrated in this mixture for 12 h at room temperature. IEF was performed on a Protean I12 IEF unit (Bio‐Rad, Hercules, California, USA) until 30 kVh were reached. Proteins were subsequently separated by SDS‐PAGE on 11.5% gels (25.5 cm × 20.5 cm; 1 mm) under the following conditions: 35 V for 1 h, 50 V for 1.5 h, and 110 V for 16.5 h at 10°C using an Ettan DALTsix electrophoresis chamber (GE Healthcare).

With these technical settings, 14 2D‐DIGE runs were conducted on frontal lobe samples from 17 AD patients and 11 matched controls, whereas 177 2D‐DIGE runs were performed on platelet samples from 124 AD patients, 61 study participants with MCI, and 168 age‐ and sex‐matched controls. When feasible, each cognitively impaired patient sample was paired with a matched control sample within the same 2D‐DIGE run.

For inter‐tissue proteomic analysis, the pooled internal standards from the frontal lobe samples and the platelet sample were used, and the internal standard labeled with Cy2 was pooled from these two internal standards of the frontal and platelet samples. These pooled standards were then separated within a single 2D‐DIGE run. To ensure reliability, this direct in‐gel comparison of the frontal lobe and platelet samples was repeated three times, with Cy3 and Cy5 labeling in dye swaps between the two tissue types in these 2D‐DIGE runs.

### 2.7 Image analysis and spot inclusion criteria

Gels were scanned with a resolution of 100 µm using a Typhoon TRIO (GE Healthcare, Uppsala, Sweden). Protein spot detection was accomplished using the DeCyder software (version 7.2, GE Healthcare); see ref. [22] for a detailed description. The standardized abundance (SA) was calculated for protein spot quantification and statistical analysis according to the manual of the DeCyder software.^38^

### 2.8 Protein identification of 2D Spots via liquid chromatography tandem mass spectrometry (LC‐MS/MS)

For mass spectrometry (MS) identification of protein spots, 250 µg of unlabeled proteins were separated under the same two‐dimensional (2D) separation conditions as the fluorescently labeled samples. However, to accommodate the higher protein load, the IEF was extended to 40 kVh at the last step to ensure adequate focusing of the proteins. After electrophoresis, the proteins were visualized with MS‐compatible silver staining.^36^

Protein spots were manually excised from the preparative 2D silver gels and destained with 15 mM potassium ferricyanide and 50 mM sodium thiosulfate pentahydrate. Reduction to break disulfide bonds was performed with 10 mM DTT in 50 mM ammonium bicarbonate buffer at 56°C for 30 min, followed by alkylation with 50 mM iodoacetamide in the same buffer for 20 min at room temperature in the dark. Gel pieces were then dehydrated with acetonitrile and dried in a speed‐vac for 20 min. Tryptic digestion was carried out overnight at 37°C with sequencing‐grade modified trypsin (12.5 ng/µL; Promega, Madison, Wisconsin, USA). Peptides were extracted in a 50% acetonitrile/5% formic acid solution using an ultrasonic bath for 15 min, then concentrated by speed‐vac for MS analysis.

For identification of the spot digests, an electrospray ionization quadrupole time‐of‐flight ([QTOF]; Compact, Bruker, Billerica, Massachusetts, USA) system coupled to an Ultimate 3000 Nano HPLC system (Dionex, Sunnyvale, California, USA) was employed. Reversed phase chromatographic separation was performed using a PepMap100 C18 trap column (300 µm × 5 mm) and a PepMap100 C18 analytical column (75 µm × 250 mm) with a flow rate of 500 nL/min. Buffers used for reverse phase chromatography were 0.1% formic acid in water and 0.08% formic acid in 80% acetonitrile/water, applied with a linear gradient over 90 min. Eluted peptides were directly sprayed into the MS, and the MS/MS spectra were interpreted using the Mascot search engine (version 2.4.1, Matrix Science, London, UK) against the Swissprot database (547,964 sequences, released in January 2016), with taxonomy restricted to *Homo sapiens* (human; 20,194 sequences). Search parameters included a mass tolerance of 10 ppm and an MS/MS tolerance of 0.1 Da. Modifications such as carbamidomethylation of cysteine, oxidation of methionine, phosphorylation of serine, threonine, and tyrosine, acetylation of lysine, and deamidation of aspartate and glutamine were allowed, with up to two missed cleavage sites. The Mascot cutoff score was set at 15, and proteins identified with two or more peptides were considered.^39^ In addition, a protein was deemed reliably identified only if its associated peptide counts were at least five times higher than those of other protein identifications from the same 2D spot. Protein identifications based on three peptides may also be accepted, provided that no peptides from other proteins are detected. However, on average, peptide counts for the primary protein identified in a 2D spot were 20‐fold higher than those for possible additional protein identifications, since the primary protein has the highest concentration in these 2D spots.

### 2.9 Western blot analysis

For one‐dimensional western blot (1D‐WB) analysis, urea‐solubilized platelet or frontal lobe samples were prepared by mixing with sample buffer (150 mM Tris‐HCl, pH 6.8, 7.5% SDS, 37.5% glycerol, and bromophenol blue), supplemented with DTT to a final concentration of 100 mM. The samples were then boiled for 4 min at 96°C and centrifuged at 20,000 × *g* for 3 min. Subsequently, 12 µg of platelet sample or 25 µg of brain sample per lane were loaded onto an 11.5% or 12.5% SDS‐PAGE gel (16 cm × 8 cm) and separated at 100 V for 150 min. A pre‐stained molecular weight (MW) marker (PageRuler Prestained Protein Ladder, 10–180 kDa; Thermo Fisher Scientific, Waltham, Massachusetts, USA) was loaded into the first and last lanes of the 1D gel. This served as a reference for monitoring protein separation by MW and observing visually the efficiency of protein transfer onto the western blot membrane.

After electrophoresis, the proteins were transferred from the 1D gels onto a polyvinylidene difluoride (PVDF) membrane (FluoroTrans W, Pall) using tank blotting at 75 V for 120 min at 10°C.

For two‐dimensional western blot (2D‐WB) analysis, 50 µg of each sample (platelet or frontal lobe) was separated based per gel as described in Section 2.6. Specific gel pieces of varying sizes, cut from the larger 25.5 cm × 20.5 cm, 11.5% SDS‐PAGE gels, were used for further analysis. Proteins from these 2D gel segments were transferred onto a PVDF membrane using tank blotting at 75 V for 90 min at 10°C.

A pre‐chilled blotting buffer at 4°C, used for both 1D and 2D gels, consisted of 25 mM Tris, 192 mM glycine, 0.05% SDS, and 20% ethanol.

A ruthenium‐based fluorescent total protein stain (RuBPS) was applied to the blotted proteins on the PVDF membranes, enabling the adjustment of any unequal protein loadings of the individual lanes of the 1D gels, thus improving the accuracy of subsequent quantification of specific antibody signals. The staining was carried out overnight at 4°C using ruthenium(II) tris(bathophenanthroline disulfonate) (RuBPS; Sigma–Aldrich, St. Louis, Missouri, USA) at a 1:100,000 dilution and scanned with a Typhoon FLA 9500 (GE Healthcare, Uppsala, Sweden) at (λ_Ex_ = 488 nm, λ_Ex_ = 610 nm).^40^

Membranes were blocked overnight at 4°C in 5% non‐fat dry milk (Bio‐Rad, Hercules) dissolved in PBS containing 0.3% Tween‐20 (termed as PBS‐T) and incubated for 2 h at room temperature with the following primary antibodies, polyclonal GSTO1 (MBS769928; MyBioSource, San Diego, California, USA) 1:4000, polyclonal SOD1 (ab13498; Abcam, USA) 1:3000, monoclonal CCS (sc‐55561; Santa Cruz Biotechnology, USA) 1:250, monoclonal GPX1 (ab108429, Abcam, USA) 1:1000. Appropriated horseradish peroxidase (HRP)–conjugated secondary antibodies (Thermo Fisher Scientific, Waltham, Massachusetts, USA) were diluted 1:20,000, and Cy5‐labeled secondary antibodies were diluted 1:5000 in PBS‐T containing 3% non‐fat dry milk and incubated for 1 h in the dark. The HRP signal was detected using an enhanced chemiluminescent substrate (FluorChem HD2, Alpha Innotech, California, USA). Antibody fluorescence signals were detected with a Typhoon FLA 9500 (GE Healthcare, Uppsala, Sweden). The 1D antibody signals of CCS and GPX1 were normalized to the RuBPS fluorescence signal and quantified with ImageQuant 8.0 software (GE Healthcare, Uppsala, Sweden).

### 2.10 TBARS assay for quantification of lipid peroxidation

Lipid peroxidation in GFP samples of AD patients and matched controls was analyzed using a modified thiobarbituric acid (TBA) test according to the method by Hossain et al.^41^ Frozen aliquots of 500 µL GFP suspension containing butylated hydroxytoluene ([BHT] at 0.2 mg/mL) to prevent further oxidation were thawed and homogenized in 5% SDS lysis buffer under sonication in an ice‐cold water bath for 10 min in the dark. Then, 1 mL of 0.4% TBA in 20% acetic acid (pH 3.5) was added, and samples were agitated with a thermomixer at 95°C for an hour, followed by cooling with tap water. One milliliter of dH₂O and 2.5 mL of n‐butanol‐pyridine (15:1 v/v) was combined with samples and shaken vigorously for 20 min. After centrifugation at 1600 × *g* for 10 min, the fluorescent compounds in the organic layer were measured using a Hitachi 850 spectrofluorometer at excitation and emission wavelengths of 515 and 553 nm, respectively. Sample TBA reactive substances (TBARS) levels were calculated using a standard curve of malondialdehyd bis(dimethyl acetal). Platelet TBARS concentrations were normalized to protein content and are thus given as pmol TBARS/µg protein.

### 2.11 *APOE* ε4 (rs429358, rs7412) and *GSTO1* (rs4925) genotyping

The genotyping procedure was performed according to Umlauf et al.^42^ In brief, genomic DNA from brain or whole blood was isolated using either the QIAamp DNA Mini Kit (Qiagen, Venlo, The Netherlands) or the PerfectPure DNA Blood Kit (Quantabio, Beverly, Massachusetts, USA) according to the manufacturer's protocols. *APOE* ε4 (rs429358, rs7412) genotyping was done according to a modified protocol adapted from Crook.^43^ Genomic DNA (15–40 ng) was amplified using *APOE‐*FW and *APOE*‐RV primers,^42^ along with kit sample buffer, 10% dimethyl sulfoxide (DMSO, 200 µM dtris‐borate‐EDTAeoxynucleotide triphosphates (dNTPs), and 0.25 units HotStarTaq Plus DNA polymerase (Qiagen, Venlo, The Netherlands). Polymerase chain reaction (PCR) was conducted on a Mastercycler EP Gradient S device (Eppendorf, Hamburg, Germany) with the following settings: 94°C for 5 min, followed by 45 cycles composed of 94°C for 20 s, 66.5°C for 30 s, and 72°C for 35 s, with a subsequent final extension at 72°C for 10 min. The PCR product was digested with HhaI (Fermentas, Waltham, Massachusetts, USA) and analyzed on a 3% tris‐borate‐EDTA (TBE:100 mmol/L Tris, 90 mmol/L boric acid, 2 mmol/L EDTA) agarose (PEQLAB Biotechnology, Erlangen, Germany) gel. Genotyping of *GSTO1* SNP rs4925 was done using an amplification‐refractory mutation PCR system.^44^ In a volume of 10 µL, 15–40 ng of genomic DNA was amplified with kit sample buffer, 200 µM dNTPs, four primers,^42^ and 0.25 units of HotStarTaq Plus DNA polymerase (Qiagen, Venlo, The Netherlands). The PCR protocol comprised an initial denaturation step at 95°C for 10 min, followed by a touch‐down PCR: 10 cycles at 95°C for 45 s, 68°C for 45 s, 72°C for 1 min 30 s; 10 cycles at 95°C for 45 s, 58°C for 45 s, 72°C for 1 min 30 s; 18 cycles at 95°C for 45 s, 54°C for 45 s, 72°C for 1 min 30 s; and 9 cycles at 95°C for 35 s, 52°C for 45 s, 72°C for 1 min, with a final extension at 72°C for 10 min. The resulting PCR fragments were analyzed on a 2.5% TBE agarose gel and were assigned to the major C or the minor A allele.

### 2.12 Statistical analysis

Only protein spots from each frontal lobe 2D‐DIGE image analysis that were at least 95% matched to the internal standard spot map of the master gel were included. This strict selection criterion resulted in 550 protein spots being included in the evaluation file of the biological variation analysis (BVA) module of the DeCyder Software. SA was then calculated for protein spot quantifications following the guidelines outlined in the DeCyder software manual^38^ and exported for further statistical analysis using either IBM SPSS Statistics 26 (SPSS Inc., Chicago, USA) or GraphPad Prism 7 (GraphPad Software, Inc., San Diego, California, USA). For statistical comparison of data of AD and matched controls, the Mann–Whitney *U* test (MWU) was applied, followed by false discovery rate (FDR) correction using the Benjamini–Hochberg method to account for multiple comparisons.^45^

To identify AD‐related platelet biomarkers, only platelet protein spots validated by MS analysis and overlapping with AD‐related protein spots from the frontal lobe 2D map were incorporated into the explorative statistical analysis. The Kruskal–Wallis H test (KWt) was applied to globally assess the significance of differences in 2D‐DIGE‐quantified platelet protein spot levels, the SA, among patients with AD, MCI, and cognitively healthy matched controls. Planned contrasts, which are predefined comparisons between the control group and the AD and MCI cohorts, were conducted using Dunn's multiple comparison test to identify protein changes between study groups. The consistency of cognition‐dependent changes in the platelet proteome was assessed based on KWt results and the direction of protein quantity changes, with a primary focus on significance among AD patients, followed by MCI patients. The general significance threshold for AD and MCI‐related protein changes was set at *p* < 0.05.

Statistical analyses of genotyping assays were performed using IBM SPSS Statistics 26. Logistic regression analysis was conducted to explore the relationship between the allele counts (*APOE* ε4 or GSTO1*C) and cognitive status (AD, MCI, or control). The exponent of the regression coefficient corresponding to the count of *APOE* ε4 alleles was taken as the odds ratio (OR) for AD and MCI patients in comparison to the cognitively healthy controls. Statistical significance was determined at *p* < 0.05, and confidence intervals (CIs) were calculated at the 95% level.

## 3 RESULTS

**FIGURE 1:**
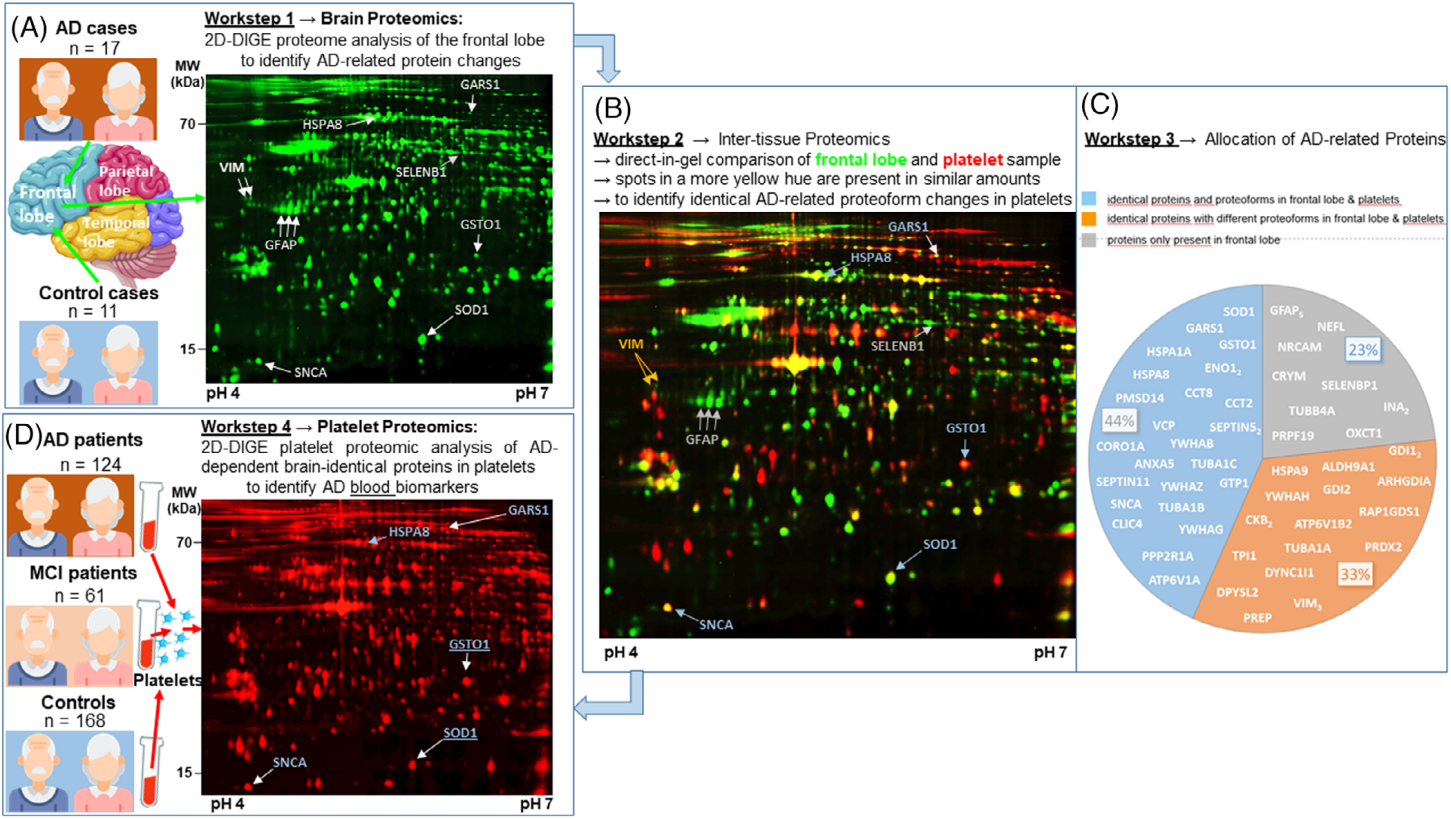
Outline of an analytical study comparing frontal lobe and platelet proteomes in Alzheimer‘s disease. (A) Characterization of AD‐related protein changes in the frontal lobe samples by 2D‐DIGE proteome analysis from 17 AD cases and 11 matched controls. A representative 2D‐DIGE image in the pH range of 4–7 is displayed. Seven of 60 significant AD‐related protein changes are highlighted (Table S4A). AD‐related protein spots were identified by MS and outlined with their respective UniProt gene names. (B) Inter‐tissue proteomics: The direct‐in‐gel comparison of the proteomes of the frontal lobe and platelet samples by 2D‐DIGE. In this 2D‐DIGE analysis, 12 µg of Cy3‐labeled pooled frontal lobe protein sample and 12 µg of Cy5‐labeled pooled platelet protein sample were separated in one analytical run by the pI (pH range 4–7) and MW. This inter‐tissue proteome analysis enables the determination of AD‐dependent protein spots (= proteoforms), which are identically present in blood platelets and frontal lobe. MS analysis was used to prove whether these protein spots from a pooled platelet protein extract had the same identity as from the frontal lobe. (C) Allocation of AD‐related proteins. The pie chart depicts 60 AD‐dependent proteoforms of the frontal lobe, which are identically present in blood platelets (blue segment, *n* = 26); proteoforms are present in platelets, but with the different pI and MW (orange segment, *n* = 20) and proteins expressed in the frontal lobe only (gray segment, *n* = 14). The subscript number next to the protein gene names indicates the number of identified proteoforms of the respective protein revealed by 2D‐DIGE and MS analysis. (D) Exploration of AD‐related changes in platelets of AD and MCI patients targeting the 26 identified AD‐dependent and brain‐identical proteoforms. A representative 2D‐DIGE platelet run is displayed, highlighting five examples from the 26 significant AD‐related protein changes observed in the frontal lobe, with an exact overlay of the corresponding proteoforms in the platelet proteome. This 2D‐DIGE analysis was performed on platelet samples from 124 AD patients, 61 MCI patients, and 168 age‐ and sex‐matched cognitively healthy controls. The results of the statistical analyses are shown in Table S4B. (A, D) Pictograms from the elderly brain, platelets, and blood tubes were procured under license from Envato. 2D‐DIGE, two‐dimensional differential gel electrophoresis; AD, Alzheimer's disease; *APOE*, apolipoprotein E; GSTO1, glutathione S‐transferase omega 1; MCI, mild cognitive impairment; MS, mass spectrometry; MW, molecular weight; pI, isoelectric point; SOD1, superoxide dismutase 1.

The main objective of this study was to identify novel platelet protein biomarkers reflecting pathophysiological processes that occur in the AD‐affected brain. Our investigations started by comparing the proteome of the frontal lobe of AD patients and matched controls (Figure 1A). Using top‐down proteome analysis via 2D‐DIGE, intact proteins can be quantified alongside the functional modifications of their proteoforms. In addition, this technology allows a direct inter‐tissue comparison between the brain and platelets within one analytical 2D gel run (Figure 1B). This approach enabled the identification of a set of brain‐identical AD‐related proteins and their proteoforms in platelets (Figure 1C). Using the same 2D‐DIGE analytical settings, these potential biomarker candidates for AD were subsequently validated in platelet samples from a second independent cohort of AD patients, individuals with MCI, and cognitively healthy controls to reveal cognition‐dependent protein profiles in the blood (Figure 1D).

### 3.1 Demographics of AD and control brain proteomics cohorts

Frontal lobe tissue samples were collected from 17 AD cases (10 men and 7 women, ages 63 to 93) and 11 matched controls (6 women and 5 men, ages 67 to 96). Subjects were closely matched according to sex and age (Table 1).

The *APOE* ε4 genotype is the strongest genetic risk factor for sporadic AD,^41^ thus being a representative quality indicator for participants recruited in an AD study. To ensure a representative study collective in a Caucasian study population, its presence should be ≈40% within the AD group and ≈15% in the cognitively healthy control group of the same age and gender.^42^ In the current study, 75% of the AD patients carry at least one *APOE* ε4 allele, significantly higher than the *APOE* ε4 allele frequency in control cases with 9% (OR = 30; *p* = 0.004; Table 1A). None of the control subjects were homozygous for the *APOE* ε4 allele, whereas 2 of 17 (12%) of patients with AD were homozygous carriers (Table 1A).

### 3.2 Demographics of AD, MCI, and control platelet proteomics cohorts

To identify peripheral blood AD biomarkers that can directly reflect molecular patterns of this disorder and detect cognitive decline as early as possible, platelet samples were collected from 124 probable AD patients with a mean age of 80 years, 61 MCI patients with a mean age of 77, and 168 matched healthy controls with a mean age of 79. Among the AD patients, 66% have at least one *APOE* ε4 allele, which is significantly higher than the frequency among control subjects with 16% (OR = 7.8; *p* = 3.5E^−15^). In the control group, only one subject was homozygous for *APOE* ε4 (1%), whereas 20% of the AD patients were homozygous carriers of the *APOE* ε4 allele. In the MCI group, 28% carried at least one *APOE* ε4 allele, which was also significantly higher than in the control group (OR = 2.0; *p* = 0.034), and 3% were homozygous for *APOE* ε4 (Table 1B). Further demographic details of the study population are listed in Table 1B.

### 3.3 Identification of proteomic changes in the frontal lobe in AD

In the study's first phase, protein extracts of the frontal lobe of 17 AD cases and 11 matched controls were analyzed to characterize AD‐related protein changes using 2D‐DIGE in the pH range of 4–7. A total of 550 protein spots were evaluated in this analysis. Sixty showed statistically significant differences in their signal intensities (each representing a specific proteoform) in the frontal lobe of AD cases. Using MS, 49 different proteins were identified from these 60 protein spots. The lower number of distinct proteins is due to the presence of multiple proteoforms of seven AD‐related proteins, including GFAP, of which all five corresponding GFAP proteoforms were significantly increased in AD cases (Tables S4A and S1; Figure S1A). All 49 proteins are also listed in the publicly available NeuroPro database,^46^ a comprehensive AD brain proteomics meta‐analysis^47^ (Table S1), further corroborating their relevance.

### 3.4 Inter‐tissue proteomics to map AD‐related changes from frontal lobe to platelets

After AD‐dependent proteoforms were identified in the frontal lobe, differently labeled pools of the frontal cortex and platelet study samples were simultaneously separated in one analytical run using the same 2D‐DIGE settings to search for identical platelet proteoforms. Such an inter‐tissue comparison on intact proteins can currently only be performed using 2D‐DIGE technology. The pooled frontal lobe extract (internal standard sample of 2D‐DIGE frontal lobe proteomics) was labeled with Cy3, displayed in green, whereas the pooled platelet sample (internal standard sample of 2D‐DIGE platelet proteomics) was labeled with Cy5, displayed in red, and both were co‐separated in one 2D‐electrophoresis run (Figure 1B).

Figure 1C illustrates which proteins and proteoforms are similar in the frontal lobe and platelets based on their isoelectric point (pI), MW, and abundance. In this case, these protein spots superimpose and appear in more yellowish tones, indicating that these proteoforms are present in quite similar amounts. Of the 60 protein spots that were found to be significantly altered in the frontal lobe of AD cases (see Section 3.3), 44% (26 protein spots) were detected at the same position on the 2D‐DIGE map of the platelet proteome, indicating potential identical functional proteoforms (Figure 1C). Subsequent MS analysis confirmed the same protein identity of these protein spots in the platelet 2D map. PlateletWeb,^48^ a database of detectable platelet proteins, has also documented the presence of these proteins. Fourteen AD‐related protein spots from the frontal lobe (23%) were not detectable in platelets, and, correspondingly, their presence was not reported in the PlateletWeb (e.g., GFAP, CRYM, and NRCAM). The abundance of the remaining 20 AD‐related protein spots (33%) of the frontal lobe was also documented in PlateletWeb.^48^ However, their proteoforms did not overlay with platelet protein spots of the 2D‐DIGE map in the pH range of 4–7. For example, various GDI1, YWHAH, TPM1, and VIM proteoforms can be detected in different regions of the platelet and frontal lobe 2D map based on their pI or MW (Figure S1A,S1B). Although many proteins of the frontal lobe are also present in platelets, their functional PTM profiles can evidently be different.

### 3.5 Search for AD‐related changes among AD‐dependent and brain‐identical proteoforms in platelets from AD and MCI patients

The 26 AD‐related altered proteoforms in the frontal lobe with identical functional proteoforms in blood platelets (see Section 3.4) were investigated for their cognition‐dependent abundance by a quantitative 2D‐DIGE platelet proteome analysis of 124 AD patients, 61 MCI patients, and 168 cognitively healthy control subjects. MCI patients were included to assess how these AD‐related brain protein levels reflect the early stage of AD in platelets (Figure 1D). The correlation factor for the FC_AD/Co_ values of the platelet protein proteoforms to the identical ones of the frontal lobe FC_AD/Co_ is r_S_ = 0.21, *p* = 0.208, whereas for the FC_MCI/Co_ values of platelet proteoforms to the identical ones of the frontal lobe FC_AD/Co_, is r_S_ = 0.24, *p* = 0.238. The correlation of the FC_AD/Co_ to FC_MCI/Co_ values of the platelet samples is moderately stronger with r_S_ = 0.44, *p* = 0.026.

Two protein spots, SOD1 and GSTO1, were consistently significantly altered in frontal lobes (Table S4A) and platelet samples of AD (Table S4B). The median platelet GSTO1 level was 40% higher in AD (*p* = 0.029) and was increased by 38% in MCI patients without meeting significance (*p* = 0.623). The canonical GSTO1 protein has been documented to be increased in the cerebellum and parietal cortex of AD cases in the NeuroPro database with a score of 4 (Table S1).

The median SOD1 levels were significantly increased in the platelets of AD patients by 8% (*p* = 0.036) and, importantly, showed an early peripheral change during cognitive decline with a 9% (*p* = 0.003) rise in MCI patients versus controls (Table S4B). In the frontal lobe of AD cases, the SOD1 increase was more pronounced, reaching 26% (*p* = 0.039).

### 3.6 Two‐dimensional western blot analysis of SOD1 and GSTO1 to identify their proteoform profiles in the frontal lobe and platelets

**FIGURE 2:**
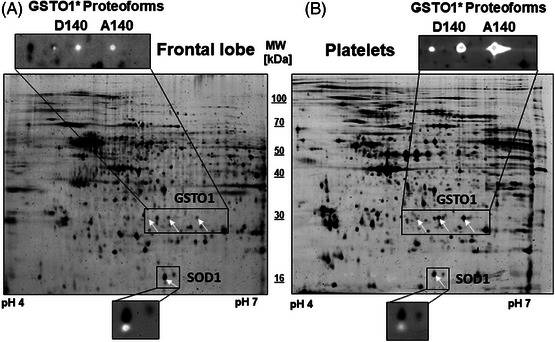
Identification of GSTO1 and SOD1 proteoforms by two‐dimensional qualitative western blot analysis in (A) the frontal lobe and (B) platelets. Thirty micrograms of Cy5‐labeled frontal lobe or platelet protein sample was applied to a 24 cm IPG strip at pH 4–7 and focused by IEF. This step was followed by a molecular weight separation on 11.5% SDS‐PAGE and transfer to a PVDF membrane. Fluorescent proteins on the PVDF membranes were scanned with a laser scanner at 650 nm wavelength and are displayed as black spots. The two large images show the 2D‐separated frontal lobe or platelet sample blotted onto the PVDF. The PVDF membrane was stained with antibodies against GSTO1 or SOD1, and the specific signals are visualized by secondary HRP‐conjugated antibodies and chemiluminescence (white spots). The small image sections depict the overlay of Cy5‐labeled 2D‐WB spot signals (black) versus antibody‐specific 2D‐WB signals (white). 2D, two‐dimensional; 2D‐WB, two‐dimensional western blot; GSTO1, glutathione S‐transferase omega 1; HRP, horseradish peroxidase; IEF, isoelectric focusing; IPG, immobilized pH gradient; kDa, kilodalton; MCI, mild cognitive impairment; MS, mass spectrometry; MW, molecular weight; pI, isoelectric point; PVDF, polyvinylidene difluoride; SOD1, superoxide dismutase 1.

Top‐down proteomics by 2D‐WB enables the identification of respective potentially multiple proteoforms of a protein within the intact proteome environment of a given tissue sample. For this purpose and to uncover possible additional proteoforms, 2D‐WB analyses were applied for SOD1 and GSTO1 using specific pan antibodies against these antigens. A single protein spot (equivalent to a proteoform) of SOD1 was detected in the 2D map of the frontal lobe and platelets at identical pI (5.8) and MW (16.5 kDa) positions (Figure 2), whereas three identical proteoforms of GSTO1 were identified in both the frontal lobe and the platelet samples (Figure 2).

**FIGURE 3:**
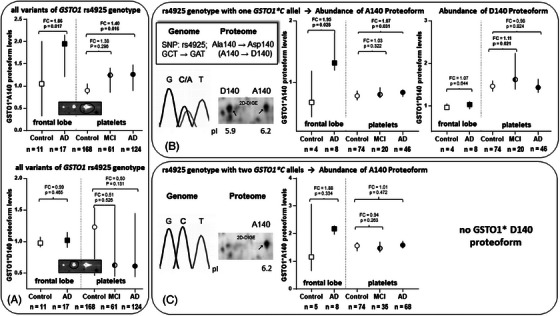
Cognition‐dependent changes in the quantity of GSTO1 proteoforms in the frontal lobe and the platelets, categorized according to the rs4925 genotype variants. The abundance of the GSTO1 proteoforms was measured by 2D‐DIGE, and the respective genotypes were determined by PCR. Proteoform levels were depicted as a median ± 95% confidence interval. Significant differences are calculated with the Mann–Whitney *U* test. (A) Abundance of the GSTO1*A140 and GSTO1*D140 proteoforms in all study groups irrespective of the rs4925 genotype variants. The small 2D gel image indicates the respective GSTO1 proteoform. (B) Abundance of the GSTO1*A140 and GSTO1*D140 proteoforms of the study individuals carrying one GSTO1*C allele. The nucleotide triplets from the DNA sequencing of the rs4924 region are shown on the left, which encode the amino acids alanine (GCT) or aspartate (GAT). Furthermore, the 2D gel section with the corresponding profile of the GSTO1 proteoforms is shown. (C) Abundance of the GSTO1*A140 proteoform of the study individuals carrying two GSTO1*C alleles and corresponding explanations as in (B). 2D‐DIGE, two‐dimensional differential gel electrophoresis; AD, Alzheimer's disease; FC, fold‐change; GSTO1, glutathione S‐transferase omega 1; MCI, mild cognitive impairment; MS, mass spectrometry; MW, molecular weight; PCR, polymerase chain reaction; pI, isoelectric point.

In a previous platelet proteomics study of AD patients, we have already characterized these three GSTO1 proteoforms and their AD‐related abundance distribution.^22^ Nonetheless, it was unexpected to discover that the same GSTO1 proteoforms also exhibit significantly increased abundance within the frontal lobe of AD cases (Figure 3). We further revealed previously that the GSTO1 spots with the pI shift from 6.2 to 5.9 correspond to the SNP rs4925. The two proteoforms with lower pI values (5.85 and 5.9) are associated with the mutant GSTO1*A genotype, resulting in the GSTO1*D140 proteoform with the main variant at pI 5.9. Neither of these proteoforms showed significant changes in AD patient platelets. The proteoform with a pI of 6.2 aligns with the major allele GSTO1*C, resulting in the proteoform GSTO1*A140.^49^ This GSTO1*A140 proteoform was increased in platelets from AD patients, with an even more substantial increase and significance observed in AD patients lacking the *APOE* ε4 allele.^22^ In our current comparative analysis, genomic DNA of brain and leukocytes was subjected to genotyping for the AD‐related SNPs rs429358 and rs7412 of *APOE* ε4 and rs4925 of *GSTO1* (Table S2). The major allele GSTO1*C (0, 1, or 2 alleles) exhibited a significant correlation with the SA of the 2D‐DIGE spots of the GSTO1*A140 proteoform in both the frontal lobe and platelets (frontal lobe: r_S_ = 0.623, *p* < 0.001; platelets: r_S_ = 0.791, *p* < 0.0001; Table S3). Accordingly, the GSTO1*D140 proteoform in platelets showed an opposite negative correlation with the allele number of the GSTO1*C genotype (platelets: r_S_ = −0.837, *p* < 0.001; Table S3), although the association was considerably weaker in the frontal lobe (frontal lobe: r_S_ = −0.212, *p* < 0.289; Table S3). Detailed distributions of *GSTO1* genotypes and respective ORs among the 17 AD and 11 control brain samples, along with the 124 AD, 61 MCI, and 168 control of the platelet cohorts, are provided in Tables 1 and S2.

Notably, the GSTO1*A140 proteoform showed a significant AD‐related increase in abundance in the frontal lobe (fold‐change [FC] = 1.86, *p* = 0.017; Figure 3A), as well as in platelets (FC = 1.40, *p* = 0.016; Figure 3A). In contrast, there was no significant change in the levels of the GSTO1*D140 proteoforms. In addition, the genotyping results confirmed that the frequency of the GSTO1*C allele count as well as the homozygote genotype GSTO1*C were higher in platelet study cohorts, especially in *APOE* ε4 allele–negative AD patients (OR = 5.2; *p* = 2.9E^−5^) and in *APOE* ε4 allele–negative MCI patients (OR = 2.2; *p* = 0.022) compared to controls (Table S2). This inverse relationship between *APOE* ε4 and homozygous GSTO1*C genotype in AD and MCI was also reflected in a slightly negative correlation (r_S_ = −0.238; *p* = 0.001). However, if cognitively healthy controls were included, this correlation is abrogated (r_S_ = −0.056; *p* = 0.296). 25; line 3).

The genetic association between *APOE* ε4 and the GSTO1*C genotype in AD was also confirmed at the proteomic level of the corresponding GSTO1*A140 proteoform in platelets. In contrast, neither a genetic nor a proteomic association was detected in the frontal lobe study cohorts (Table S2 and Figure S2). However, the number of *APOE* ε4–negative AD cases was limited (*n* = 4), which may not be fully representative.

Due to these contradictory results, the dependence of the GSTO*A140 proteoform and the corresponding rs4925 SNP distribution in AD was investigated in more detail. For this purpose, the levels of this proteoform with the same GSTO1*C allele count were compared separately between AD, MCI patients, and control subjects in both frontal lobes and platelets. Of interest, this split showed that the GSTO1*A140 proteoform was AD‐dependently increased in the frontal lobe and platelets within subjects with one GSTO1*C allele. Therefore, this AD‐dependent proteoform increase is also independent of its allele frequency, although weaker in the platelets (Figure 3B). No higher amount of the GSTO1*A140 proteoform was observed in the platelets of the MCI patients (Figure 3B). Of interest, the GSTO1*A140 platelet proteoform was not AD‐dependently altered within subjects carrying two GSTO1*C alleles but still had a trend to be increased in the frontal lobe (Figure 3C).

The top‐down 2D‐DIGE method enabled us to find out that the major SNP‐dependent proteoform, GSTO1*A140, exhibits identical alterations in both the frontal lobes and platelets. Furthermore, our findings show that both genetic and non‐genetic factors can contribute to the elevated levels of the GSTO1*A proteoform in frontal lobes and platelets in AD. Nevertheless, the genetic predominance of the homozygous GSTO1*C genotype could not be substantiated in this limited cohort of AD brain autopsy cases.

### 3.7 Identification of functionally integrated proteins for SOD1 and GSTO1 expressed similarly in the frontal lobe and platelets

**FIGURE 4:**
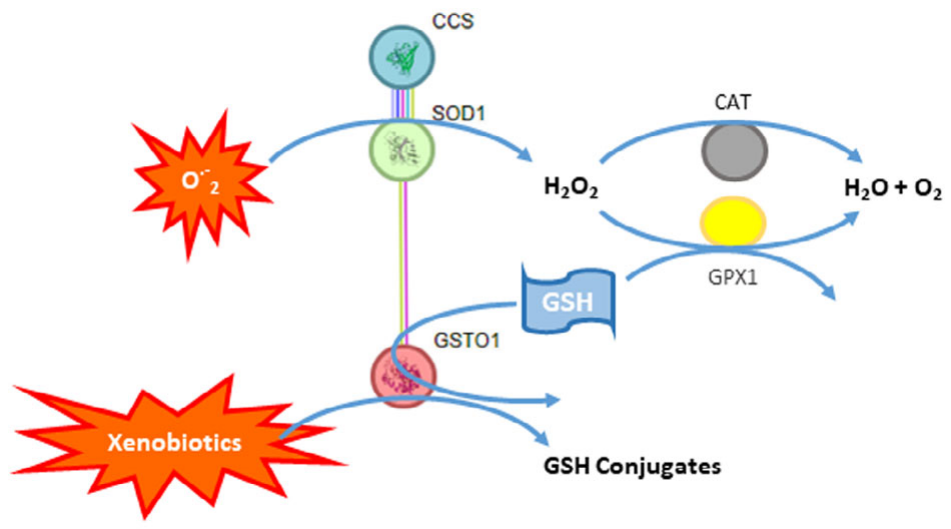
Functional proteome interaction analysis of identical AD‐related proteins in the frontal lobe and platelets. Physical direct functional proteome association analysis of the AD‐related proteins SOD1 and GSTO1 was made with the STRING database analysis tool. Colored linear slopes indicate the identified way of interaction; pink was experimentally determined, blue was from curated databases, and yellow was text mining. By STRING analysis, cellular oxidant detoxification was the most significantly enriched GO Biological Process (*p* = 0.0016). In addition, textbook knowledge and scientific literature have further implemented that several members of the GPX family have essential functional cooperation with SOD1 and GSTO1 in antioxidative cell protection. A Human Protein Atlas and PlateletWeb database search revealed that GPX1 is the only GPX family member present in both brain and platelets. CAT, catalase; CCS, copper chaperone for superoxide dismutase; GSH, glutathione; GPX1, glutathione peroxidase 1; GSTO1, glutathione S‐transferase omega 1; O.‐2, superoxide radical anions; SOD1, superoxide dismutase 1.

The next objective was to determine if additional functional integrative proteins for SOD1 and GSTO1 are expressed similarly in the frontal lobe and platelets and, if so, whether they exhibit consistent AD‐related changes in both biological sample types. A literature review and functional network analysis were performed to identify synergistic functions of SOD1 and GSTO1 in the cell's antioxidant defense mechanism, including their directly interacting proteins (Figure 4). SOD1 converts superoxide anion radicals into H_2_O_2_, which is then processed to water and oxygen either by GPX or catalase.^50^ Together with SODs, GPXs, and glutathione S‐transferases (GSTs), they are the most crucial enzymes in the cellular antioxidant system.^51^ The protein family of GSTs catalyzes the conjugation of glutathione to a wide range of electrophilic metabolites of xenobiotics for detoxification.^52^ Seleno‐dependent GPXs, akin to their protein family members, the seleno‐independent GSTs, utilize reduced glutathione as a substrate to catalyze the breakdown of H_2_O_2._ ^53^ GPX1 is the only member of the large GPX family (GPX1‐GPX7) being expressed in brain and platelets.^48^ Another candidate was identified by STRING pathway analysis involving the strong physical interaction between CCS and SOD1. CCS is crucial for activating the SOD1 enzyme by supplying copper as a cofactor.^54^ CCS has been detected in both the brain and platelets.^48^ Based on these database research findings, GPX1 and CCS were selected for subsequent proteomic analyses to identify additional biomarkers reflecting AD‐dependent frontal lobe changes in platelets.

### 3.8 Qualitative and quantitative assessments of CCS and GPX1 levels in the frontal lobe and platelets according to cognitive status

First, 2D‐WB analysis of frontal lobe and platelet proteome samples was performed to determine the presence as well as the pI and the MW of CCS and GPX1 protein spots. These proteome analyses also enabled us to find the position of CCS and GPX1 in the corresponding 2D‐DIGE maps. For CCS, a spot with a pI of 5.4 and an MW of 29 kDa was observed, consistently detected at identical positions in both platelets and the frontal lobe proteome (Figure S3; white arrows). The GPX1 antibody recognized two protein spots at the same position in each sample with a pI of 5.8 or 6.1 and an MW of 29 kDa. MS analysis confirmed the CCS 2D‐WB signals in frontal lobe and platelet samples. Of the two GPX1 western blot signals, the one with a pI of 5.8 and an MW of 29 kDa could be confirmed as GPX1 in the frontal lobe and platelets by MS identification (Figure S3; depicted with white arrows). Regarding the other 2D western GPX1 signals (light blue arrows), MS analysis failed to identify GPX1‐specific peptides in either tissue sample (Figure S3). Most likely, the protein amounts of GPX1 in these spots are below the detection limit of MS analyses, provided that the respective antibody has a higher sensitivity than the MS approach, or there are false‐positive cross‐reactions of the antibody.

**FIGURE 5:**
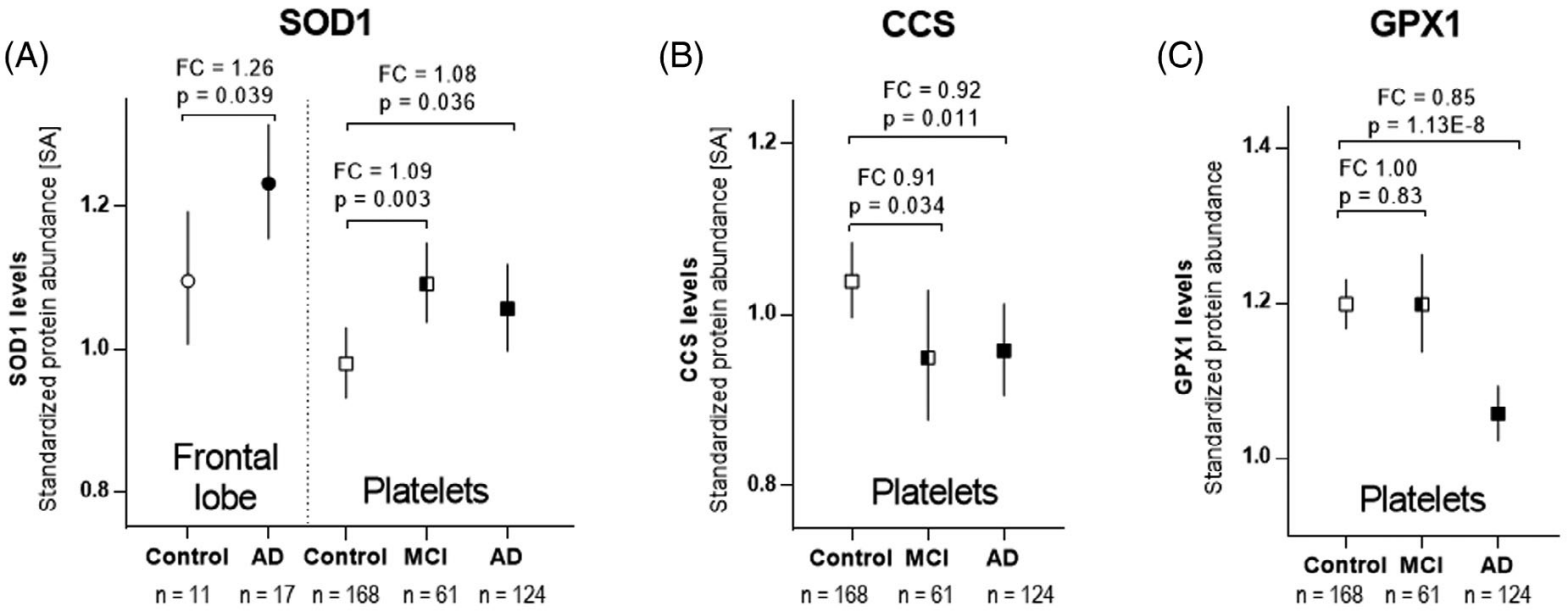
Protein abundance of SOD1, CCS, and GPX1 in the frontal lobe or platelets in relation to the cognitive status. The abundance of the (A) SOD1, (B) CCS, and (C) GPX1 proteoforms were quantified by 2D‐DIGE analysis. Proteoform levels are depicted as a median ± 95% confidence interval. Median FCs are calculated from the SA of each particular protein spot. The significance of changes (*p*‐values) were calculated between the AD or MCI and control groups using the Mann–Whitney *U* test. 2D‐DIGE, two‐dimensional differential gel electrophoresis; AD, Alzheimer's disease; CCS– copper chaperone for superoxide dismutase; FC, fold‐change; GPX1, glutathione peroxidase 1; GSTO1, glutathione S‐transferase omega 1; MCI, mild cognitive impairment; SA, standardized abundance; SOD1, superoxide dismutase 1.

The 2D‐WB and MS‐identified 2D spots were then assigned and evaluated in the current 2D‐DIGE analysis of AD frontal lobe and platelet proteome samples. The CCS (Figure S4A,S4B) and GPX1 spot signals (Figure S5A,S5B) were relatively faint but could be well evaluated in the 2D‐DIGE platelet analysis. Although platelet SOD1 levels were increased significantly in AD and MCI patients (FC_AD/Co _= 1.08; *p* = 0.036 and FC_MCI/Co _= 1.09; *p* = 0.003; Figure 5A), the platelets of patients with AD and MCI had significantly lower CCS levels compared to the cognitively healthy control group (FC_AD/Co _= 0.92; *p* = 0.011 and FC_MCI/Co _= 0.91; *p* = 0.034; Figure 5B). Moreover, AD patients exhibited significantly lower amounts of GPX1 than the control group (FC_AD/Co _= 0.85; *p* = 1.13E^−8^; Figure 5C), whereas the levels in MCI patients remained unchanged (Figure 5C). However, the CCS and GPX1 spots were weaker in the 2D‐DIGE frontal lobe analysis compared to the platelet signals (Figures S4B and S5B), making reliable matching from all 2D‐DIGE runs difficult. Thus, to get stronger signals for CCS and GPX1, a 1D‐WB analysis of these proteins from frontal lobe samples was carried out with a higher load (of 25 µg) than the previously applied 12.5 µg for 2D‐DIGE analysis. Moreover, it can be expected that the CCS and GPX1 antibodies have a high affinity for their respective antigens and may, therefore, give stronger signals than the CCS and GPX1 CyDye spot signals.1D‐WB analyses of CCS and GPX1 were also made on a randomly selected set of platelet samples from AD patients (*n* = 15) and controls (*n* = 15) for further validations.

**FIGURE 6:**
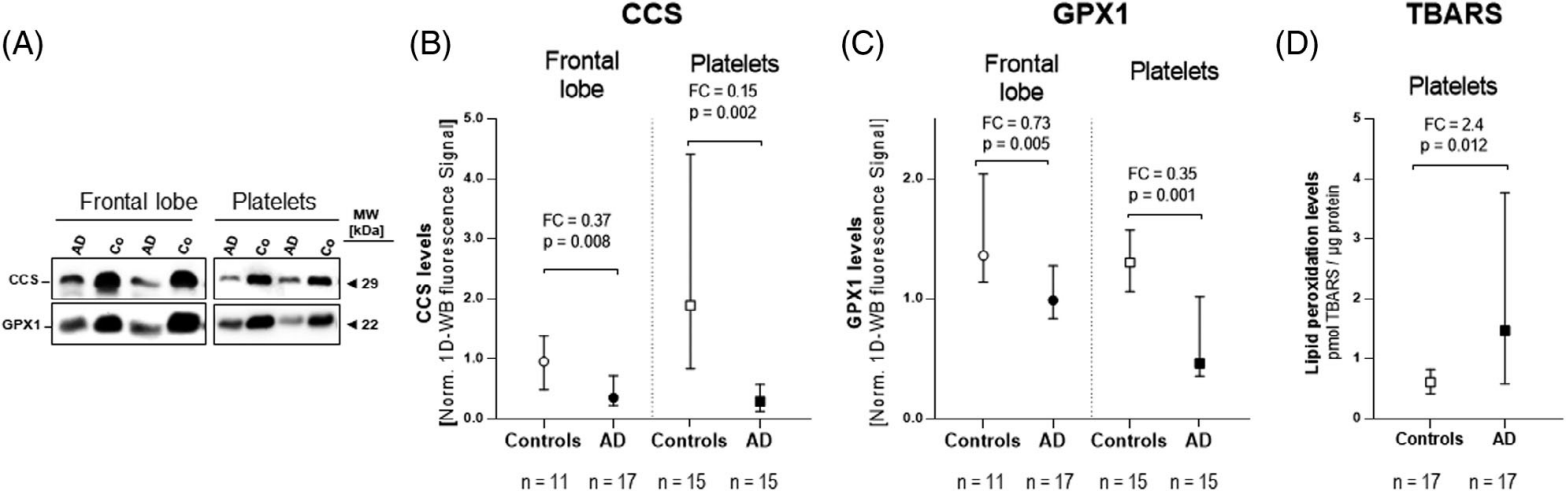
Quantitative analysis of CCS and GPX1 levels in frontal lobe and platelets by 1D western blot analysis and lipid peroxidation in platelets of AD patients and controls. (A) Representative western blot shows CCS and GPX1 protein abundance levels in two AD patients and two control subjects in the frontal lobe and platelets of AD. Thirty micrograms of platelet and 50 µg of frontal lobe protein extract were separated according to their MW on 11.5% acrylamide gel SDS‐PAGE (10 cm separation distance). Ruthenium‐based whole protein stain of blotted proteins on the PVDF membrane was performed to normalize and, accordingly, to adjust to equal protein loads per sample for CCS and GPX1 quantification. Images of the 1D‐WB, including the ruthenium stain of blotted proteins, are shown in Figures S4 and S5. Median FCs are calculated from the normalized 1D‐WB Cy5 fluorescence signals of the CCS protein bands and the GPX1 protein bands. (D) Lipid peroxidation by TBARS (thiobarbituric acid reactive substances) was quantified from gel‐filtered platelets and was normalized to the amount of the respective platelet protein used. Normalized levels of (B) CCS, (C) GPX1, and (D) TBARS were depicted as a median ± 95% confidence interval. Median FCs are calculated from the normalized values of each particular protein band. The significances of the changes (*p*‐values) were calculated between the AD and control groups using the Mann–Whitney *U* test. AD, Alzheimer's disease; CCS, copper chaperone for superoxide dismutase; FC, fold‐change; GPX1, glutathione peroxidase 1; GSTO1, Glutathione S‐transferase omega 1; kDa, kilo Dalton; MCI, mild cognitive impairment; MW, molecular weight; SOD1, superoxide dismutase 1; TBARS, thiobarbituric acid reactive substances; PVDF, polyvinylidene difluoride.

Well‐detectable signals of CCS at 29 kDa and GPX1 at 22 kDa in frontal lobe and platelet samples were achieved (Figure 6A). Quantitative evaluations of these 1D‐WB signals confirmed significantly lower amounts of CCS and GPX1 in the frontal lobes of AD cases (CCS: FC_AD/Co _= 0.37; *p* = 0.008 and GPX1: FC_AD/Co _= 0.73; *p* = 0.005; Figure 6B,C) and AD platelets (CCS: FC_AD/Co _= 0.15; *p* = 0.002 and GPX1: FC_AD/Co _= 0.35; *p* = 0.001; Figure 6B,C).

To assess how changes in these antioxidant proteins impact oxidative stress in platelets, samples from 17 patients with AD and 17 age‐ and sex‐matched controls were investigated for lipid peroxidation using the TBARS assay. The median platelet TBARS content in AD patients (1.47 pmol/µg protein) was significantly (*p* = 0.012) higher than observed in the control group (0.61 pmol/µg protein). This represents a 2.4‐fold increase compared to matched controls (Figure 6D). This is in line with a previous study reporting a significant elevation in TBARS levels in the frontal cortex of AD and MCI cases.^55^

## 4 DISCUSSION

Blood‐based biomarker tools are urgently needed to improve AD diagnosis. Blood platelets have been shown to reflect several AD‐related brain changes. This study aimed to identify protein alterations in the frontal lobe associated with AD that are identically present in platelets from AD patients. Given the predominant alterations of proteoforms such as Aß and p‐tau in AD, a top‐down proteomics approach using 2D‐DIGE technology was applied. This method of analyzing intact proteins is unique for unbiased proteoform detection, as demonstrated here by the identification of AD‐ and SNP‐dependent proteoforms of GSTO1. In total, four AD‐dependent changes in the antioxidant system in both the frontal lobe and platelets were revealed; two of them are already altered in MCI.

Initially, 60 AD‐dependent protein changes were identified in the frontal lobe using 2D‐DIGE, providing a basis for identifying identical proteoform‐based AD biomarker candidates in platelets. To validate the brain proteome results from this small AD study, they were compared with the NeuroPro database,^46^ a comprehensive AD brain proteomics meta‐analysis,^47^ as shown in Table S1. The NeuroPro database compiles data from 38 bottom‐up AD proteome studies, featuring 18,119 proteins from various brain regions, 848 of which have been identified as altered in AD.^46^ In our current study, 40 of the 49 AD‐identified frontal lobe–related protein changes are also listed in the NeuroPro database as significantly altered. The consistency with the NeuroPro database is high: 89% of the proteins changed in the same direction, including GFAP, ANXA5, and NEFL (Table S1).

Bottom‐up proteomics, as used in the NeuroPro meta‐analysis, evaluates canonical proteins such as APP and microtubule‐associated protein tau (MAPT), since all analysis steps must be performed on the peptide mixtures of digested protein samples. Thus, in the NeuroPro database, both canonical proteins, APP and MAPT, were consistently found to be increased. However, conventional bottom‐up proteomics derives its results from the incidental origin of APP peptides of Aβ fragments in amyloid plaques and intracellular APP levels in neurons or other cell types of usually not micro‐dissected brain tissue samples. Consequently, bottom‐up proteomics cannot differentially quantify the actual amounts of intact APP protein and its proteoforms, such as Aβ fragments. The same limitation applies to MAPT and its hyperphosphorylated peptides from neurofibrillary tangles. Without knowledge of increased Aβ and p‐tau levels, unbiased bottom‐up proteomics studies can only show elevated levels of canonical APP and MAPT proteins, failing to indicate the altered cleavage or hyperphosphorylation of their proteoforms.

Thus, the AD and SNP rs4925–dependent alteration of one of the three GSTO1 proteoforms, identified herein by 2D‐DIGE due to a pI shift caused by an alanine‐to‐aspartate mutation, cannot be detected using conventional bottom‐up proteomics. This proteoform of the major SNP rs4925 variant is increased in the frontal lobe and platelets in AD; in the latter, this was particularly evident in the *APOE* ε4–negative AD patients.^22^

A genetic study showed previously that the minor variant of SNP rs4925 in GSTO1 was linked to a delayed onset of AD by 7 years and Parkinson's disease by 9 years.^56^, ^57^ This suggests a protective role of SNP rs4925 and is consistent with our findings of enrichment of the major allele GSTO1*C in AD patients. However, subsequent studies could not confirm this association.^58^, ^59^, ^60^

Of interest, when using a simple and brief neuropsychological test such as the commonly used MMSE to examine age‐ and sex‐matched controls for an AD study, no significant increase in the major allele GSTO1*C was found in either AD patients or patients with MCI.^42^ In contrast, when a more precise neuropsychological test battery, such as a CERAD‐Plus, was used, ≈40% of these age‐ and gender‐matched controls were diagnosed as MCI, and a significant enrichment of the GSTO1*C allele was detected in both the AD and MCI patients compared to the cognitively healthy control group. Likewise, the OR of the reference SNP *APOE* ε4 increased from 3 to 7 in the AD study cohort.^42^

In total, 26 brain‐identical proteoforms were found in platelets from the 60 AD‐dependent changes in the frontal lobe using a 2D‐DIGE analysis setup, in which the proteome of the differentially labeled frontal lobe and platelet protein samples were compared simultaneously in one run. A subsequent platelet proteome analysis of 124 AD, 61 MCI patients, and 168 matched controls using the same 2D‐DIGE settings revealed two brain‐identical AD‐dependent alterations in platelets: GSTO1*A140 and SOD1, with SOD1 also consistently altered in MCI. According to the NeuroPro meta‐analysis,^46^, ^47^ SOD1 has also been found consistently to be elevated in the frontal cortex of AD cases with a score of 9 (Table S1). An integrative brain‐to‐CSF proteomics study revealed that elevated SOD1 levels in AD brains are reflected in the CSF,^61^ and another study found a significant correlation between SOD1 and p‐tau levels in CSF.^62^

Of interest, increased enzymatic activity of SOD in the erythrocytes of AD patients,^17^, ^63^ as well as elevated SOD1 messenger RNA (mRNA) levels in peripheral mononuclear cells of both AD and MCI patients, have been observed.^64^ Elevated SOD1 levels in AD may be due to increased production of superoxide radical anions, which in turn leads to increased production of H_2_O_2_. If not adequately balanced by GPX or catalase, this potentially leads to the generation of hydroxyl radicals. This imbalance is particularly critical in the brain, which is highly susceptible to oxidative stress.^6^ Given that oxidative stress worsens neurodegeneration in AD and reduced glutathione and antioxidant system disturbances are key contributors, components like GPXs have been studied extensively in AD.^65^

A bioinformatics‐supported search revealed GPX1 and CCS as closely interacting functional proteins of GSTO1 and SOD1, which are similarly present in the brain and platelets. 2D‐WB analysis confirmed identical GPX1 and CCS proteoforms in the frontal lobe and platelets. Proteomic analyses of the frontal lobe and platelet study samples showed reduced GPX1 and CCS levels in AD as well as decreased CCS levels in MCI patients.

Contrary to our results of decreased GPX1 levels, the NeuroPro meta‐analysis reports consistently increased GPX1 levels in the frontal cortex.^46^ However, targeted studies measuring GPX activity in mitochondria and synaptosomes have also shown reduced enzymatic activity in the frontal cortex of AD cases, which was less pronounced in MCI.^55^ In addition, reduced GPX activity and protein levels have been demonstrated consistently in blood components such as plasma,^66^, ^67^ serum,^68^ diluted whole blood,^69^ and erythrocytes^70^, ^71^ in AD patients. Some of these studies report an increase^70^ or no change^72^ in GPX blood levels. These conflicting results highlight potential bias in AD biomarker research and the need for better standardization, improved analytical techniques, and more validation studies. Nonetheless, the reduced GPX1 levels in platelets uncovered in the present study represent a promising new blood biomarker reflecting identical antioxidant changes in the frontal cortex in AD.

Alterations in CCS levels in AD are largely unknown, except for a bottom‐up proteomics study listed in NeuroPro, which identified decreased CCS in the frontal cortex among numerous other AD‐related changes.^73^ CCS deficiency has been linked to increased Aß production in a SHSY5Y neuroblastoma model.^74^ CCS regulates cellular copper levels; excess copper bound to CCS is transported via XIAP to the 26S proteasome for elimination.^75^ AD patients exhibit systemic changes in copper metabolism and altered brain copper distribution, leading to copper accumulation in Aß plaques and deficiencies in certain brain regions.^76^

Both SOD1 and CCS showed significant changes in the platelets of AD and MCI patients, suggesting their potential as early diagnostic markers for AD. Although increased SOD has been reported in the brain and blood, this is the first study linking alterations in SOD1 and CCS in platelets to early cognitive decline in AD. The rise in SOD1 and drop in its cofactor CCS offer new insights into the dysfunction of the antioxidant defense system in AD.

Although this discovery‐oriented and highly selective frontal lobe‐to‐platelet comparison proteomics study exclusively identified significant alterations in the antioxidant system, it was limited in identifying markers well‐known in AD. No significant alterations in APP or MAPT could be detected here in AD by 2D‐DIGE analysis of the frontal cortex and platelets. The current 2D‐DIGE settings, optimized for the typical pI and MW range of 2D electrophoresis, could not detect the high MW of APP (135–110 kDa) and the small Aβ fragments (4.3 kDa for Aβ1‐40 and 4.5 kDa for Aβ1‐42). Several limitations should be noted when conducting a top‐down proteomics study using 2D‐DIGE analysis. This technique struggles to detect low‐abundance or high‐MW proteins. Moreover, we only examined the pH range 4–7, leaving the 6–9 range, which includes AD biomarkers like MAOB,^15^ yet unexplored. In addition, 2D‐DIGE analysis is time intensive and lacks automation in many workflow steps.^23^ Other limitations of this study include the extended time interval between patients' deaths and the post‐mortem collection of frontal lobe samples, as well as the fact that the blood samples were not obtained from the same individuals as the brain samples.

In conclusion, this study uses a unique inter‐tissue top‐down proteomic approach to identify novel peripheral platelet proteoform‐based blood biomarkers for AD, exhibiting changes identical to those observed in the AD‐affected brain. It is important to note that these alterations were found in a SNP‐based proteoform of GSTO1 and the concentrations of other antioxidant proteins, including GPX1, SOD1, and CCS, in the frontal lobe and platelets of AD patients; the latter two were also found changed in the platelets of MCI. Validating and integrating these platelet markers with traditional AD blood biomarkers, such as Aβ, tau, and *APOE* ε4, in larger cohorts may accelerate the development of blood tests for causal factors, risk assessment, and treatment guidance.

## CONFLICT OF INTEREST STATEMENT

The authors declare no conflict of interest. Author disclosures are available in the Supporting Information.

## CONSENT STATEMENT

The medical ethics committee of Vienna approved the blood sampling study. All participants and/or their legal representatives provided written informed content for participation.

## DATA AVAILABLITY STATEMENT

The proteomics raw data of this study are available in Mendeley Data at (https://data.mendeley.com/datasets/kjhsnpff5k/1).

## Supporting information



Supporting Information

Supporting Information

Supporting Information
