# Supplementary material for: Platelets mirror changes in the frontal lobe antioxidant system in Alzheimer's disease
Source: Alzheimers Dement. 2025 Apr 6;21(4):e70117. doi: 10.1002/alz.70117 (PMC11972982; doi:10.1002/alz.70117)
Supplement: Supplementary file 2 — Supporting Information [file ALZ-21-e70117-s001.pptx]

## Slide 1
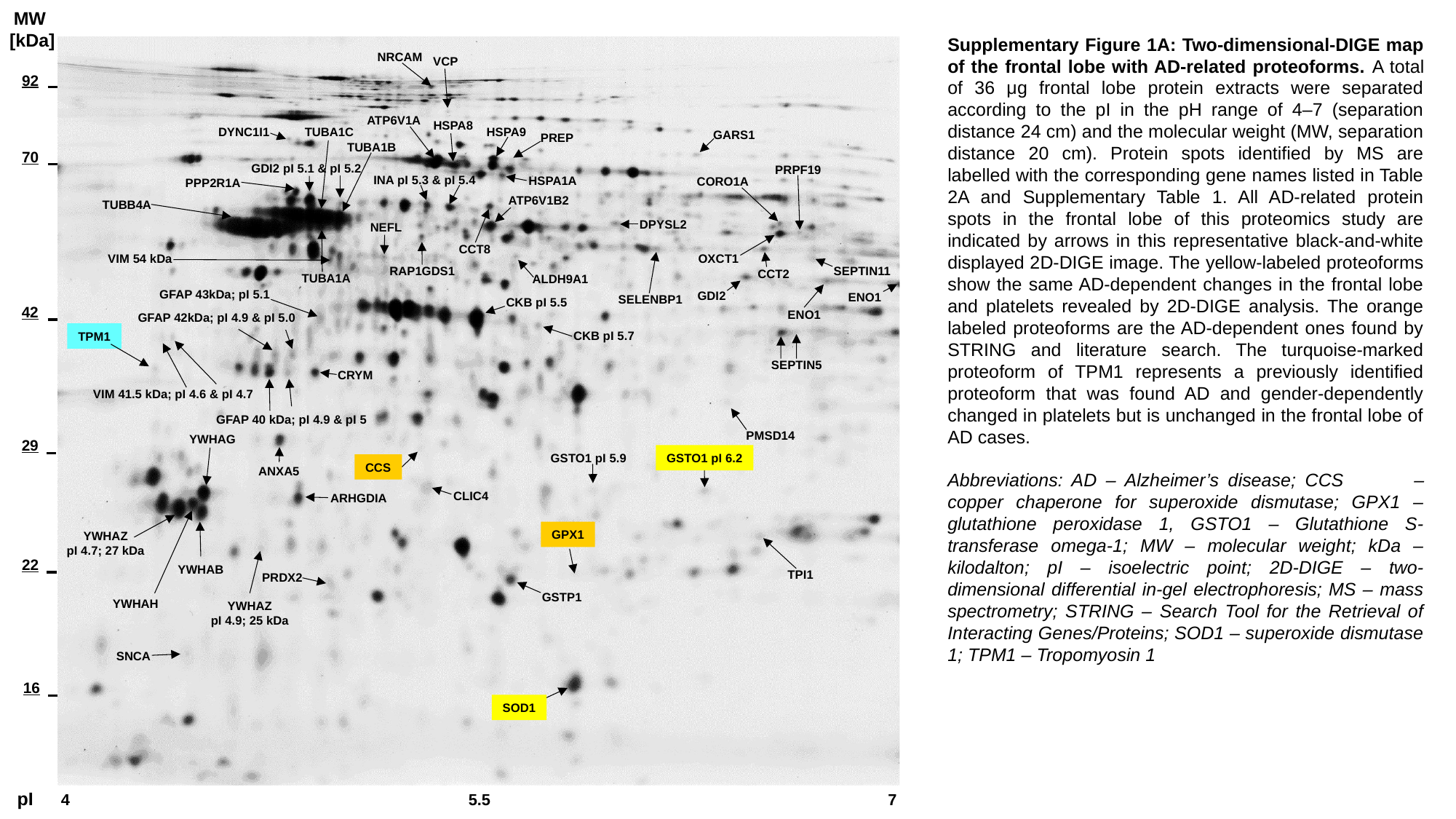

MW
[kDa]
Supplementary Figure 1A: Two-dimensional-DIGE map of the frontal lobe with AD-related proteoforms. A total of 36 μg frontal lobe protein extracts were separated according to the pI in the pH range of 4–7 (separation distance 24 cm) and the molecular weight (MW, separation distance 20 cm). Protein spots identified by MS are labelled with the corresponding gene names listed in Table 2A and Supplementary Table 1. All AD-related protein spots in the frontal lobe of this proteomics study are indicated by arrows in this representative black-and-white displayed 2D-DIGE image. The yellow-labeled proteoforms show the same AD-dependent changes in the frontal lobe and platelets revealed by 2D-DIGE analysis. The orange labeled proteoforms are the AD-dependent ones found by STRING and literature search. The turquoise-marked proteoform of TPM1 represents a previously identified proteoform that was found AD and gender-dependently changed in platelets but is unchanged in the frontal lobe of AD cases.
Abbreviations: AD – Alzheimer’s disease; CCS	 – copper chaperone for superoxide dismutase; GPX1 – glutathione peroxidase 1, GSTO1 – Glutathione S-transferase omega-1; MW – molecular weight; kDa – kilodalton; pI – isoelectric point; 2D-DIGE – two-dimensional differential in-gel electrophoresis; MS – mass spectrometry; STRING – Search Tool for the Retrieval of Interacting Genes/Proteins; SOD1 – superoxide dismutase 1; TPM1 – Tropomyosin 1
NRCAM
VCP
ATP6V1A
HSPA8
DYNC1I1
TUBA1C
HSPA9
GARS1
PREP
TUBA1B
GDI2 pI 5.1 & pI 5.2
PRPF19
INA pI 5.3 & pI 5.4
HSPA1A
CORO1A
PPP2R1A
ATP6V1B2
TUBB4A
DPYSL2
NEFL
CCT8
VIM 54 kDa
OXCT1
RAP1GDS1
SEPTIN11
CCT2
TUBA1A
ALDH9A1
GFAP 43kDa; pI 5.1
GDI2
ENO1
SELENBP1
CKB pI 5.5
ENO1
GFAP 42kDa; pI 4.9 & pI 5.0
CKB pI 5.7
SEPTIN5
CRYM
VIM 41.5 kDa; pI 4.6 & pI 4.7
GFAP 40 kDa; pI 4.9 & pI 5
PMSD14
YWHAG
GSTO1 pI 5.9
GSTO1 pI 6.2
CCS
ANXA5
CLIC4
ARHGDIA
GPX1
YWHAZ
pI 4.7; 27 kDa
YWHAB
TPI1
PRDX2
GSTP1
YWHAH
YWHAZ
pI 4.9; 25 kDa
SNCA
SOD1
92
70
42
TPM1
29
22
16
pI
4
5.5
7

## Slide 2
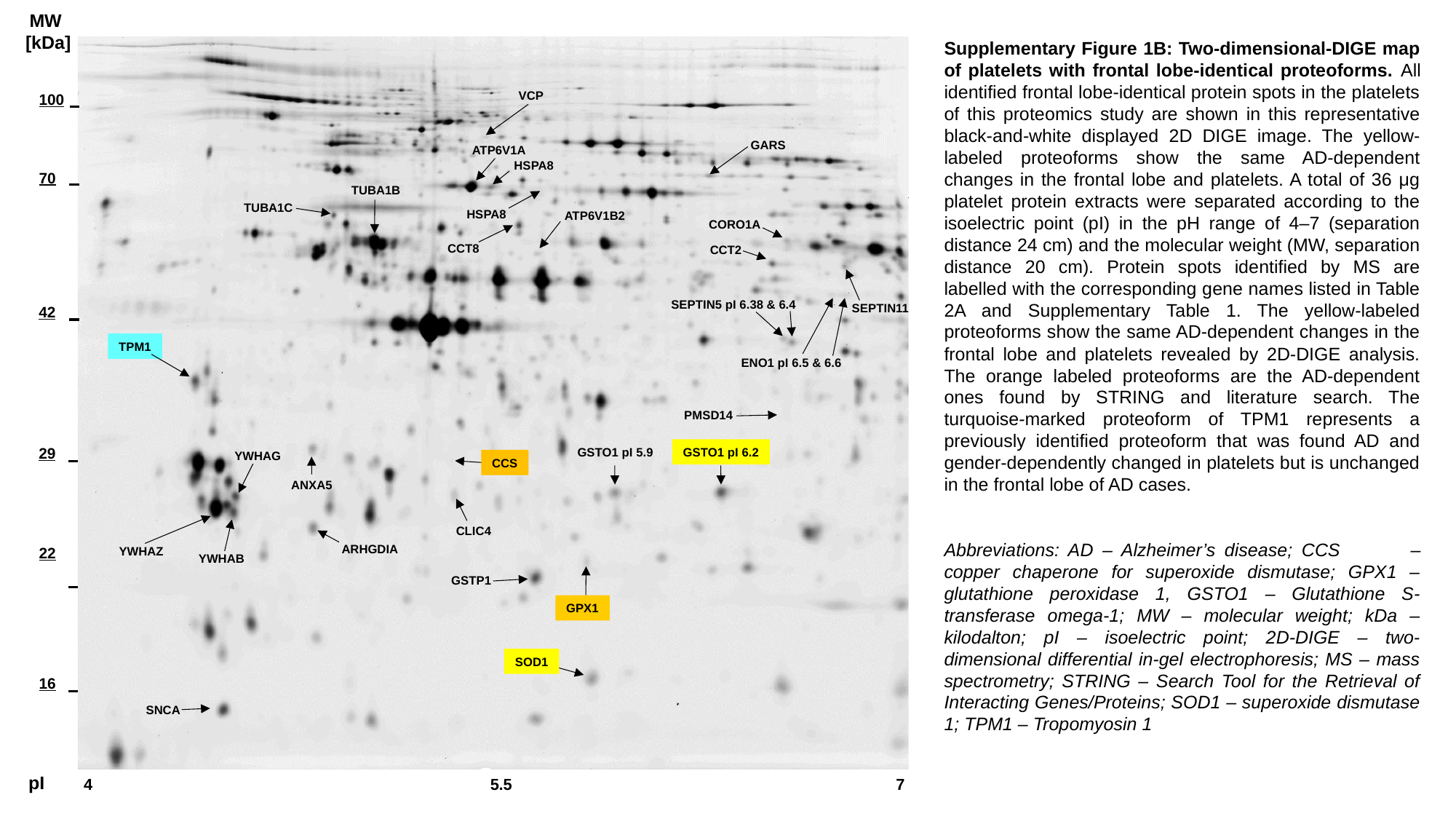

MW
[kDa]
Supplementary Figure 1B: Two-dimensional-DIGE map of platelets with frontal lobe-identical proteoforms. All identified frontal lobe-identical protein spots in the platelets of this proteomics study are shown in this representative black-and-white displayed 2D DIGE image. The yellow-labeled proteoforms show the same AD-dependent changes in the frontal lobe and platelets. A total of 36 μg platelet protein extracts were separated according to the isoelectric point (pI) in the pH range of 4–7 (separation distance 24 cm) and the molecular weight (MW, separation distance 20 cm). Protein spots identified by MS are labelled with the corresponding gene names listed in Table 2A and Supplementary Table 1. The yellow-labeled proteoforms show the same AD-dependent changes in the frontal lobe and platelets revealed by 2D-DIGE analysis. The orange labeled proteoforms are the AD-dependent ones found by STRING and literature search. The turquoise-marked proteoform of TPM1 represents a previously identified proteoform that was found AD and gender-dependently changed in platelets but is unchanged in the frontal lobe of AD cases.
Abbreviations: AD – Alzheimer’s disease; CCS	 – copper chaperone for superoxide dismutase; GPX1 – glutathione peroxidase 1, GSTO1 – Glutathione S-transferase omega-1; MW – molecular weight; kDa – kilodalton; pI – isoelectric point; 2D-DIGE – two-dimensional differential in-gel electrophoresis; MS – mass spectrometry; STRING – Search Tool for the Retrieval of Interacting Genes/Proteins; SOD1 – superoxide dismutase 1; TPM1 – Tropomyosin 1
VCP
GARS
ATP6V1A
HSPA8
TUBA1B
TUBA1C
HSPA8
ATP6V1B2
CORO1A
CCT8
CCT2
SEPTIN5 pI 6.38 & 6.4
SEPTIN11
ENO1 pI 6.5 & 6.6
PMSD14
GSTO1 pI 5.9
GSTO1 pI 6.2
YWHAG
CCS
ANXA5
CLIC4
ARHGDIA
YWHAZ
YWHAB
GSTP1
GPX1
SOD1
SNCA
100
70
42
TPM1
29
22
16
pI
4
5.5
7

## Slide 3
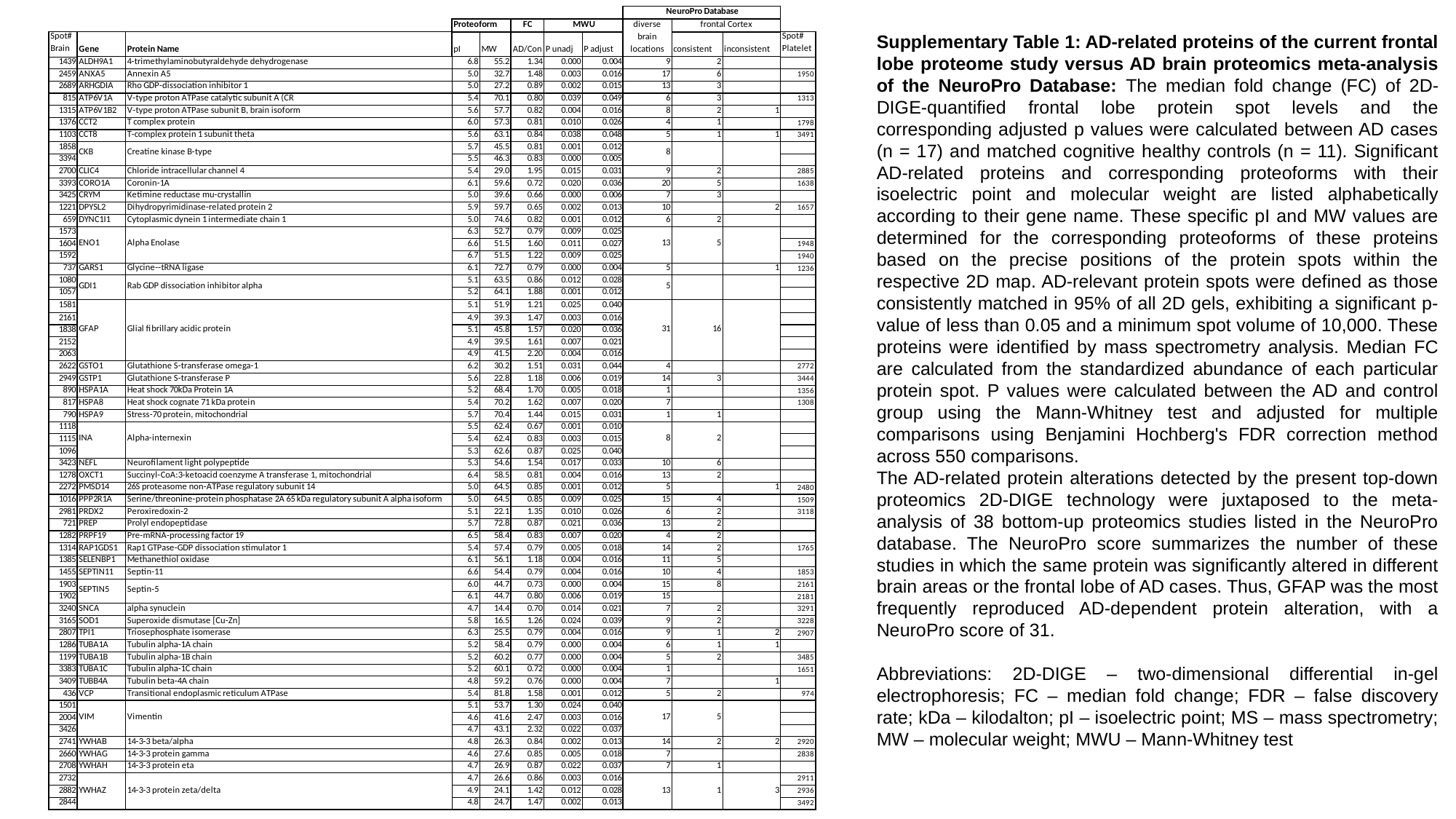

Supplementary Table 1: AD-related proteins of the current frontal lobe proteome study versus AD brain proteomics meta-analysis of the NeuroPro Database: The median fold change (FC) of 2D-DIGE-quantified frontal lobe protein spot levels and the corresponding adjusted p values were calculated between AD cases (n = 17) and matched cognitive healthy controls (n = 11). Significant AD-related proteins and corresponding proteoforms with their isoelectric point and molecular weight are listed alphabetically according to their gene name. These specific pI and MW values are determined for the corresponding proteoforms of these proteins based on the precise positions of the protein spots within the respective 2D map. AD-relevant protein spots were defined as those consistently matched in 95% of all 2D gels, exhibiting a significant p-value of less than 0.05 and a minimum spot volume of 10,000. These proteins were identified by mass spectrometry analysis. Median FC are calculated from the standardized abundance of each particular protein spot. P values were calculated between the AD and control group using the Mann-Whitney test and adjusted for multiple comparisons using Benjamini Hochberg's FDR correction method across 550 comparisons.
The AD-related protein alterations detected by the present top-down proteomics 2D-DIGE technology were juxtaposed to the meta-analysis of 38 bottom-up proteomics studies listed in the NeuroPro database. The NeuroPro score summarizes the number of these studies in which the same protein was significantly altered in different brain areas or the frontal lobe of AD cases. Thus, GFAP was the most frequently reproduced AD-dependent protein alteration, with a NeuroPro score of 31.
Abbreviations: 2D-DIGE – two-dimensional differential in-gel electrophoresis; FC – median fold change; FDR – false discovery rate; kDa – kilodalton; pI – isoelectric point; MS – mass spectrometry; MW – molecular weight; MWU – Mann-Whitney test

## Slide 4
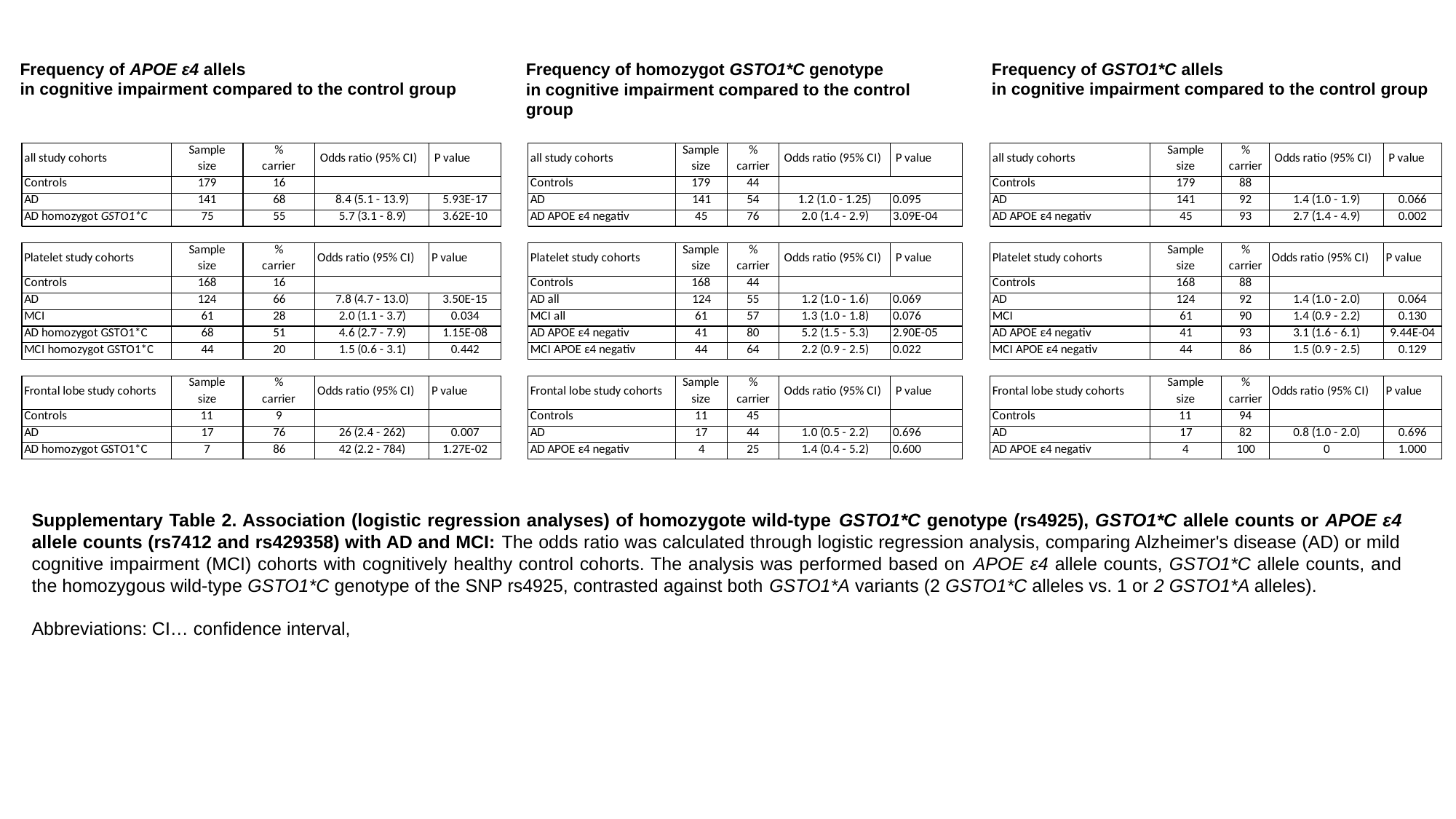

Frequency of APOE ε4 allels
in cognitive impairment compared to the control group
Frequency of homozygot GSTO1*C genotype
in cognitive impairment compared to the control group
Frequency of GSTO1*C allels
in cognitive impairment compared to the control group
Supplementary Table 2. Association (logistic regression analyses) of homozygote wild-type GSTO1*C genotype (rs4925), GSTO1*C allele counts or APOE ε4 allele counts (rs7412 and rs429358) with AD and MCI: The odds ratio was calculated through logistic regression analysis, comparing Alzheimer's disease (AD) or mild cognitive impairment (MCI) cohorts with cognitively healthy control cohorts. The analysis was performed based on APOE ε4 allele counts, GSTO1*C allele counts, and the homozygous wild-type GSTO1*C genotype of the SNP rs4925, contrasted against both GSTO1*A variants (2 GSTO1*C alleles vs. 1 or 2 GSTO1*A alleles).
Abbreviations: CI… confidence interval,

## Slide 5
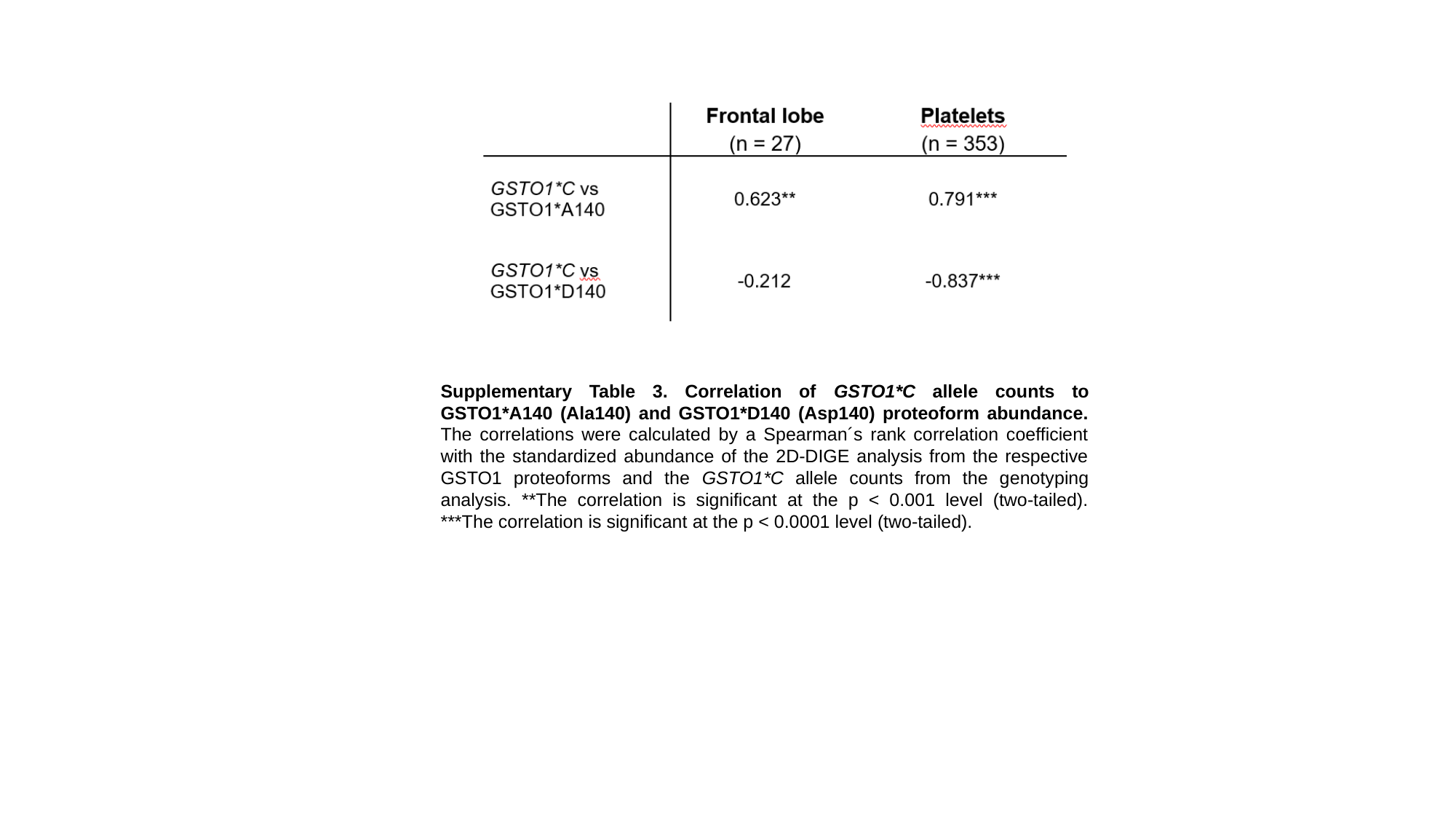

Supplementary Table 3. Correlation of GSTO1*C allele counts to GSTO1*A140 (Ala140) and GSTO1*D140 (Asp140) proteoform abundance. The correlations were calculated by a Spearman´s rank correlation coefficient with the standardized abundance of the 2D-DIGE analysis from the respective GSTO1 proteoforms and the GSTO1*C allele counts from the genotyping analysis. **The correlation is significant at the p < 0.001 level (two-tailed). ***The correlation is significant at the p < 0.0001 level (two-tailed).

## Slide 6
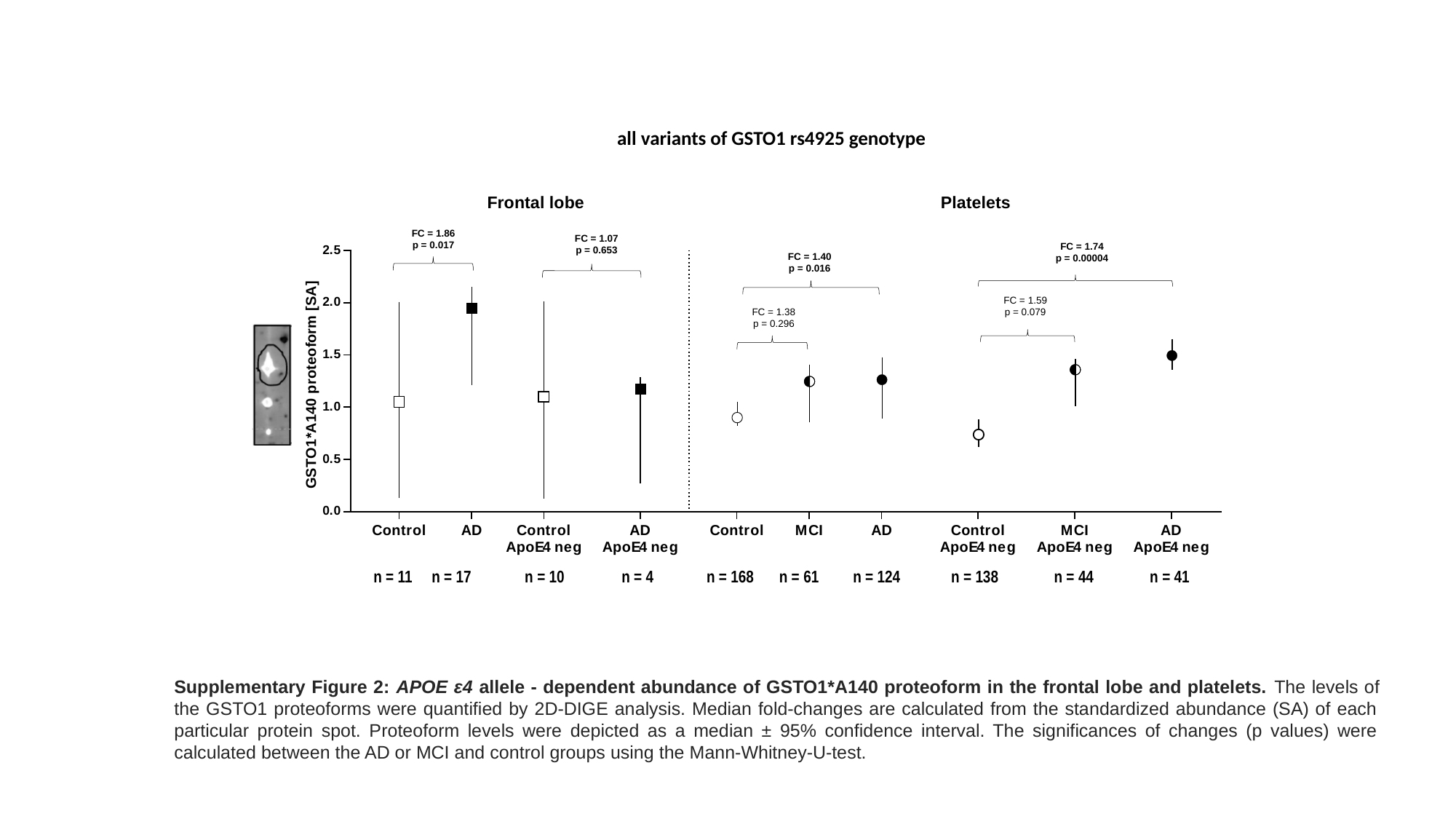

all variants of GSTO1 rs4925 genotype
Frontal lobe
Platelets
FC = 1.86
p = 0.017
FC = 1.07
p = 0.653
FC = 1.74
p = 0.00004
FC = 1.40
p = 0.016
FC = 1.59
p = 0.079
FC = 1.38
p = 0.296
n = 11
n = 17
n = 10
n = 4
n = 168
n = 61
n = 124
n = 138
n = 44
n = 41
Supplementary Figure 2: APOE ε4 allele - dependent abundance of GSTO1*A140 proteoform in the frontal lobe and platelets. The levels of the GSTO1 proteoforms were quantified by 2D-DIGE analysis. Median fold-changes are calculated from the standardized abundance (SA) of each particular protein spot. Proteoform levels were depicted as a median ± 95% confidence interval. The significances of changes (p values) were calculated between the AD or MCI and control groups using the Mann-Whitney-U-test.

## Slide 7
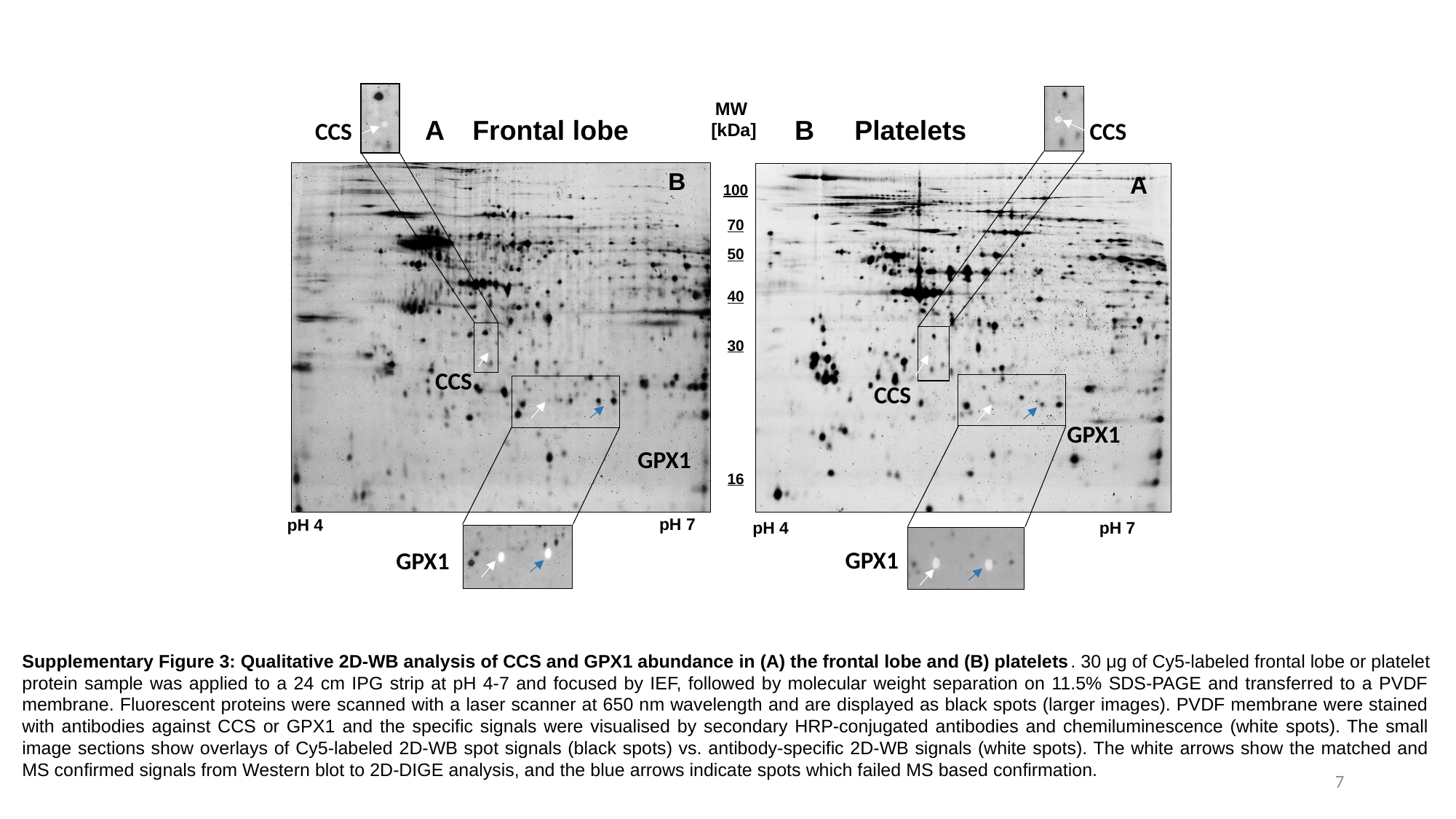

MW
[kDa]
100
70
50
40
30
16
A
Frontal lobe
B
Platelets
CCS
CCS
B
A
CCS
CCS
GPX1
GPX1
pH 7
pH 4
pH 7
pH 4
GPX1
GPX1
Supplementary Figure 3: Qualitative 2D-WB analysis of CCS and GPX1 abundance in (A) the frontal lobe and (B) platelets. 30 μg of Cy5-labeled frontal lobe or platelet protein sample was applied to a 24 cm IPG strip at pH 4-7 and focused by IEF, followed by molecular weight separation on 11.5% SDS-PAGE and transferred to a PVDF membrane. Fluorescent proteins were scanned with a laser scanner at 650 nm wavelength and are displayed as black spots (larger images). PVDF membrane were stained with antibodies against CCS or GPX1 and the specific signals were visualised by secondary HRP-conjugated antibodies and chemiluminescence (white spots). The small image sections show overlays of Cy5-labeled 2D-WB spot signals (black spots) vs. antibody-specific 2D-WB signals (white spots). The white arrows show the matched and MS confirmed signals from Western blot to 2D-DIGE analysis, and the blue arrows indicate spots which failed MS based confirmation.
7

## Slide 8
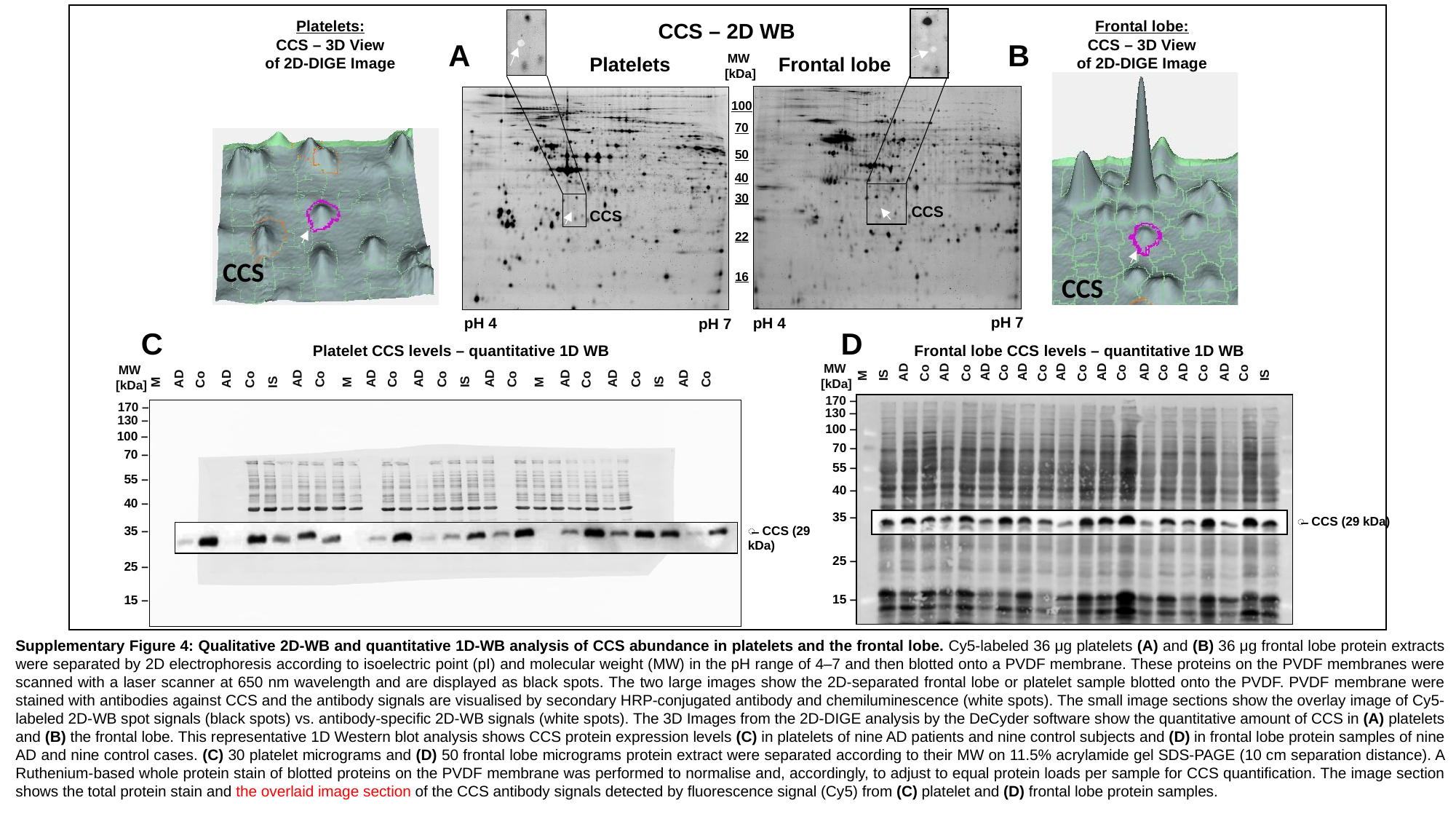

Platelets:
CCS – 3D View
of 2D-DIGE Image
Frontal lobe:
CCS – 3D View
of 2D-DIGE Image
CCS – 2D WB
A
B
MW
[kDa]
Platelets
Frontal lobe
100
70
50
40
30
CCS
CCS
22
CCS
16
CCS
pH 7
pH 4
pH 4
pH 7
C
D
Platelet CCS levels – quantitative 1D WB
Frontal lobe CCS levels – quantitative 1D WB
MW
[kDa]
MW
[kDa]
AD
AD
AD
AD
AD
AD
AD
AD
AD
Co
Co
Co
Co
Co
Co
Co
Co
Co
AD
AD
IS
AD
AD
IS
AD
AD
AD
AD
AD
Co
M
Co
Co
Co
Co
Co
Co
Co
Co
IS
IS
IS
M
M
M
170 –
170 –
130 –
130 –
100 –
100 –
70 –
70 –
55 –
55 –
40 –
40 –
35 –
̶ CCS (29 kDa)
̶ CCS (29 kDa)
35 –
25 –
25 –
15 –
15 –
Supplementary Figure 4: Qualitative 2D-WB and quantitative 1D-WB analysis of CCS abundance in platelets and the frontal lobe. Cy5-labeled 36 μg platelets (A) and (B) 36 μg frontal lobe protein extracts were separated by 2D electrophoresis according to isoelectric point (pI) and molecular weight (MW) in the pH range of 4–7 and then blotted onto a PVDF membrane. These proteins on the PVDF membranes were scanned with a laser scanner at 650 nm wavelength and are displayed as black spots. The two large images show the 2D-separated frontal lobe or platelet sample blotted onto the PVDF. PVDF membrane were stained with antibodies against CCS and the antibody signals are visualised by secondary HRP-conjugated antibody and chemiluminescence (white spots). The small image sections show the overlay image of Cy5-labeled 2D-WB spot signals (black spots) vs. antibody-specific 2D-WB signals (white spots). The 3D Images from the 2D-DIGE analysis by the DeCyder software show the quantitative amount of CCS in (A) platelets and (B) the frontal lobe. This representative 1D Western blot analysis shows CCS protein expression levels (C) in platelets of nine AD patients and nine control subjects and (D) in frontal lobe protein samples of nine AD and nine control cases. (C) 30 platelet micrograms and (D) 50 frontal lobe micrograms protein extract were separated according to their MW on 11.5% acrylamide gel SDS-PAGE (10 cm separation distance). A Ruthenium-based whole protein stain of blotted proteins on the PVDF membrane was performed to normalise and, accordingly, to adjust to equal protein loads per sample for CCS quantification. The image section shows the total protein stain and the overlaid image section of the CCS antibody signals detected by fluorescence signal (Cy5) from (C) platelet and (D) frontal lobe protein samples.

## Slide 9
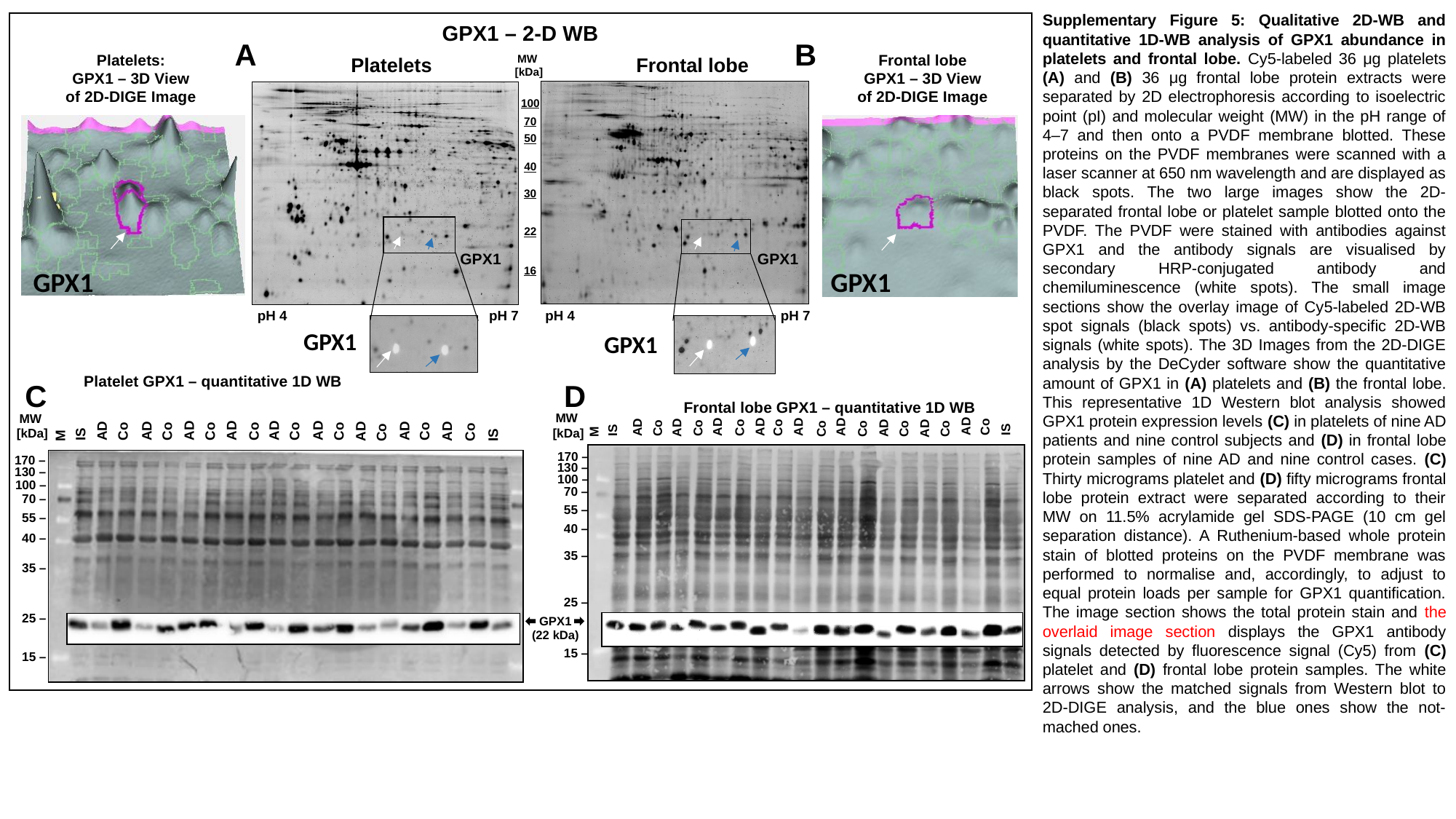

Supplementary Figure 5: Qualitative 2D-WB and quantitative 1D-WB analysis of GPX1 abundance in platelets and frontal lobe. Cy5-labeled 36 μg platelets (A) and (B) 36 μg frontal lobe protein extracts were separated by 2D electrophoresis according to isoelectric point (pI) and molecular weight (MW) in the pH range of 4–7 and then onto a PVDF membrane blotted. These proteins on the PVDF membranes were scanned with a laser scanner at 650 nm wavelength and are displayed as black spots. The two large images show the 2D-separated frontal lobe or platelet sample blotted onto the PVDF. The PVDF were stained with antibodies against GPX1 and the antibody signals are visualised by secondary HRP-conjugated antibody and chemiluminescence (white spots). The small image sections show the overlay image of Cy5-labeled 2D-WB spot signals (black spots) vs. antibody-specific 2D-WB signals (white spots). The 3D Images from the 2D-DIGE analysis by the DeCyder software show the quantitative amount of GPX1 in (A) platelets and (B) the frontal lobe. This representative 1D Western blot analysis showed GPX1 protein expression levels (C) in platelets of nine AD patients and nine control subjects and (D) in frontal lobe protein samples of nine AD and nine control cases. (C) Thirty micrograms platelet and (D) fifty micrograms frontal lobe protein extract were separated according to their MW on 11.5% acrylamide gel SDS-PAGE (10 cm gel separation distance). A Ruthenium-based whole protein stain of blotted proteins on the PVDF membrane was performed to normalise and, accordingly, to adjust to equal protein loads per sample for GPX1 quantification. The image section shows the total protein stain and the overlaid image section displays the GPX1 antibody signals detected by fluorescence signal (Cy5) from (C) platelet and (D) frontal lobe protein samples. The white arrows show the matched signals from Western blot to 2D-DIGE analysis, and the blue ones show the not-mached ones.
GPX1 – 2-D WB
A
B
Platelets:
GPX1 – 3D View
of 2D-DIGE Image
Frontal lobe
GPX1 – 3D View
of 2D-DIGE Image
MW
[kDa]
100
70
50
40
22
16
pH 4
pH 7
pH 4
pH 7
Platelets
Frontal lobe
30
GPX1
GPX1
GPX1
GPX1
GPX1
GPX1
Platelet GPX1 – quantitative 1D WB
C
D
Frontal lobe GPX1 – quantitative 1D WB
MW
[kDa]
MW
[kDa]
AD
AD
AD
AD
AD
AD
Co
AD
AD
Co
Co
AD
Co
Co
Co
Co
Co
Co
AD
AD
AD
AD
AD
AD
AD
AD
AD
Co
Co
Co
Co
Co
Co
Co
Co
Co
IS
IS
M
IS
IS
M
170 –
170 –
130 –
130 –
100 –
100 –
70 –
70 –
55 –
55 –
40 –
40 –
35 –
35 –
25 –
25 –
GPX1
(22 kDa)
15 –
15 –
