# Supplementary material for: Platelets mirror changes in the frontal lobe antioxidant system in Alzheimer's disease
Source: Alzheimers Dement. 2025 Apr 6;21(4):e70117. doi: 10.1002/alz.70117 (PMC11972982; doi:10.1002/alz.70117)
Supplement: Supplementary file 3 — Supporting Information [file ALZ-21-e70117-s002.docx]

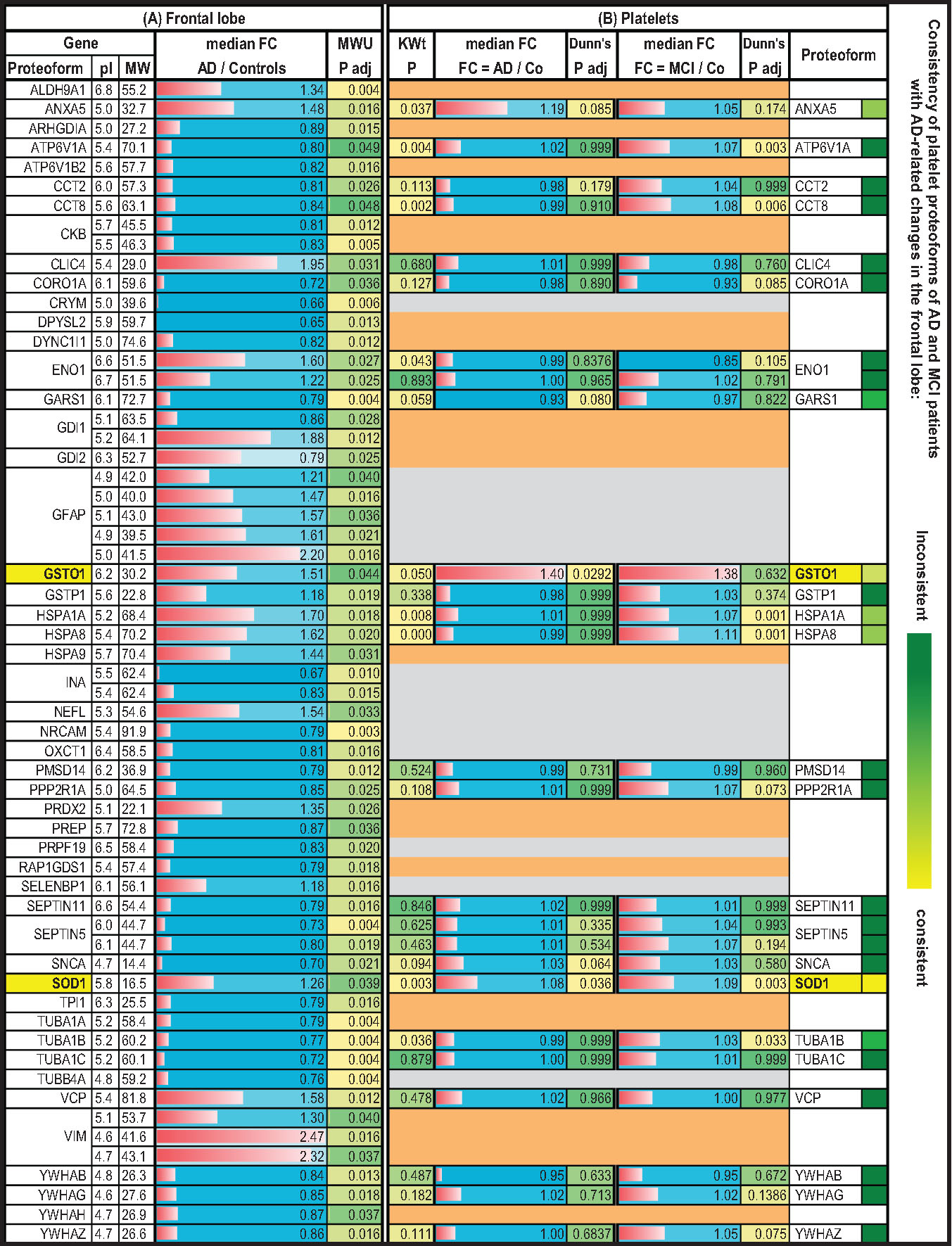


**Supplementary Table 4 (A)** Alzheimer's disease-related protein changes in the frontal lobe: 2D-DIGE analyses were conducted on samples from 17 AD cases and 11 matched cognitively healthy controls. Protein spots (= proteoforms) that consistently matched in 95% of all 2D gels and had a minimum spot volume of 10,000 were included in the statistical evaluation. Statistical comparison was done with Mann-Whitney-U-test. P-values were adjusted for multiple comparisons using Benjamini Hochberg's FDR correction method across 550 protein spot comparisons. Median FCs were calculated from the SA of each particular protein spot. These AD-related proteins were identified by mass spectrometry. Significant AD-related proteins and corresponding proteoforms are listed alphabetically according to their gene name. Actual pI and MW are given for the respective proteoforms of these proteins based on the position of the protein spot in the respective 2D map. **(B)** Characterisation of AD-related frontal lobe-identical proteoform changes in blood platelets by statistical evaluation of their AD and mild cognitive impairment dependency compared to cognitively healthy control subjects: Kruskal-Wallis H test (KWt; p < 0.05) indicates the significance of variance of 2D-DIGE-quantified platelet protein spot levels of the particular proteins and proteoforms between patients with AD (n = 124), MCI (n = 61) and matched cognitive healthy controls (n = 168). Followed by a planned contrast with a Dunn's multiple comparison test the control group was compared against the cohorts of patients with AD and MCI for significant changes (Dunn's adjusted p < 0.05). The degree of consistency of these cognition-dependent changes in the platelet proteome was indicated by the significance of the KWt - p values and the same direction of changes in protein abundance, followed primarily by the significance in the AD patients and subsequently by the significance in the MCI patients. The lines labelled in orange indicate identical proteins with different proteoforms in the frontal lobe, and the lines labelled in grey indicate proteins only present in the frontal lobe.

*Abbreviations: 2D-DIGE two-dimensional differential gel electrophoresis, AD – Alzheimer’s disease, adj – adjusted , APOE – Apolipoprotein E, FC – fold-change, FDR – false discovery rate, GSTO1 – Glutathione S-transferase omega-1, KWt – Kruskal-Wallis H test – KWt, MCI – mild cognitive impairment, MS – mass spectrometry, MW – molecular weight, pI – isoelectric point, SA – standardized abundance*
